# Extracellular vesicles mediate stem cell signaling and systemic RNAi in planarians

**DOI:** 10.1126/sciadv.ady1461

**Published:** 2026-02-06

**Authors:** Vidyanand Sasidharan, Laura Ancellotti, Viraj Doddihal, Carolyn Brewster, Frederick Mann, Mary Cathleen McKinney, Joseph Varberg, Eric Ross, Fengyan Deng, Kexi Yi, Alejandro Sánchez Alvarado

**Affiliations:** ^1^ Stowers Institute for Medical Research, Kansas City, MO, USA.; ^2^ Howard Hughes Medical Institute, Chevy Chase, MD, USA.; ^3^ Charles Perkins Center, School of Medical Sciences, University of Sydney, Sydney, Australia.; ^4^ Institute of Science and Technology Austria, Am Campus 1, Klosterneuburg 3400, Austria.

## Abstract

Planarian flatworms are known for their remarkable regenerative capacity; however, the precise intercellular communication mechanisms underlying this process remain unsolved. Here, we report the discovery and characterization of abundant extracellular vesicles (EVs) in planarians. Using imaging and molecular analysis, we show conservation of biogenesis, morphology, and protein composition of planarian EVs. Environmental stressors significantly elevate EV release, indicating that planarians dynamically regulate vesicle production. Functionally, planarian EVs mediate intercellular communication by transferring regulatory signals: We find that they shuttle small RNAs that effect systemic RNA interference (RNAi) throughout the organism. Notably, gene knockdown experiments reveal a crucial role for AGO-3, a member of the Argonaute family of proteins, in modulating the association of small interfering RNAs with EVs, linking the intracellular RNAi machinery to EV-based signaling. These findings highlight EVs as pivotal mediators of cell-cell communication in planarians, with broad implications for understanding the coordination of gene regulation and tissue regeneration in animals.

## Teaser

Planarian extracellular vesicles carry small RNAs that mediate systemic gene silencing and coordinate regeneration.

## INTRODUCTION

In many organisms, traumatic events such as amputation and inflammation can trigger a complex biological response known as regeneration, through which damaged tissues are restored to health. The animal kingdom exhibits a wide range of regenerative capabilities (*1*). At one end of the spectrum, some animals can only heal wounds without restoring missing tissues. At the other end, certain animals can completely regenerate lost body parts (*2*) or even regenerate an entire organism from an amputated part (*3*). Several factors, including chemical and mechanical signals, play a role in regeneration (*4*, *5*). The cellular agents driving regeneration in adult tissues both respond to and produce these signals (*1*). The chemical signals are part of the secretome, a complex mixture of biomolecules that includes two main fractions: soluble components and vesicular components. The soluble fraction, released directly into the extracellular space, comprises proteins, growth factors, cytokines, and other biomolecules. In contrast, the vesicular fraction consists of membrane-enclosed structures like exosomes, microvesicles, and apoptotic bodies, which contain nucleic acids, proteins, and metabolites. Secreted vesicles can modulate target cell activity by interacting with cell membranes or releasing their contents into the cytoplasm, influencing processes such as wound healing (*6*), angiogenesis (*7*), neurite outgrowth (*8*), and inflammatory responses (*9*). Despite the well-understood role of the soluble secretome in regeneration, the role of the vesicular secretome remains poorly understood.

Extracellular vesicles (EVs) are a key component of the cellular secretome, comprising a diverse group of lipid bilayer–enclosed nanoparticles secreted by cells into the extracellular space. These vesicles, which from nano- to microsizes, play crucial roles in intercellular communication and various biological processes. The three primary subtypes of EVs—exosomes, microvesicles (ectosomes), and apoptotic bodies—are distinguished on the basis of their biogenesis, release pathway, size, content, and function (*10*). The discovery of EVs dates back to 1967 when P. Wolf described them as “platelet dust” and noted their role in blood coagulation (*11*). Subsequently, Pan and Johnstone provided the first electron micrograph of EVs isolated from maturing reticulocytes (*12*), and in 1987, Johnstone coined the term “exosomes” for vesicles shed by cells. Today, it is recognized that EVs are ubiquitously released by cells under both normal and pathological conditions and can be found in various bodily fluids, including blood, urine, and saliva (*13*). Exosomes, typically 30 to 150 nm in size, originate from late endosomes, often involving the endosomal sorting complexes required for transport (ESCRT) machinery, and are formed by the inward budding of multivesicular bodies (MVBs) before being released into the extracellular space upon fusion with the plasma membrane. Microvesicles, which range from 100 to 1000 nm, are derived from plasma membrane budding and require cytoskeleton components, molecular motors, and fusion machinery for their formation (*14*). The largest EVs, apoptotic bodies (1 to 5 μm), are produced during programmed cell death. Despite substantial progress, the complete mechanisms of EV biogenesis remain incompletely understood. Recent research has identified additional EV subtypes, such as autophagic EVs, stress EVs, and matrix vesicles (*15*), highlighting the diverse landscape and functions of EVs that require further exploration.

Upon secretion, EVs disperse omnidirectionally, interacting with and modulating the functions of surrounding cells (*16*). EVs contain a diverse range of biomolecules, including proteins, nucleic acids [mRNA, microRNA (miRNA), and DNA], lipids, metabolites, and surface molecules (*14*). The composition of EVs reflects their cell of origin and varies depending on the physiological state of the cell and its microenvironment (*17*). For instance, some EVs are enriched with proteins like Rab GTPases, SNAREs, and Annexins, which are crucial for MVB formation (*18*, *19*). Others are enriched in proteins involved in cytokinesis and ribosomal functions (*20*). By facilitating the transfer of bioactive molecules between cells, EVs mediate intercellular communication and influence a range of physiological and pathological processes (*17*).

EVs play pivotal roles in many biological processes, including immune system regulation, tissue repair, and cell maturation (*21*). However, EVs also contribute to the progression of diseases such as cancer, neurodegenerative disorders, and cardiovascular conditions. In cancer, EVs can spread malignancy by transferring oncogenic biomolecules to healthy cells (*22*), and they have been used as a prognostic tool for cancer diagnosis (*23*). In the context of neurodegenerative diseases like Parkinson’s, EVs facilitate the transfer of misfolded α-synuclein proteins between cells, potentially contributing to neurodegeneration (*24*). Conversely, EVs have been explored as therapeutic agents. For example, EVs derived from mesenchymal stem cells (MSC-EVs) and neural stem cells (NSC-EVs) have shown promise in improving neuropathological conditions and aiding recovery from behavioral and cognitive deficits associated with neurological injuries and disorders (*9*, *25*). In addition, EVs have been found to enhance mammalian tissue regeneration in vitro. However, mammalian models have limited regenerative capabilities, and in vivo studies focusing on EV biogenesis and other biological functions are scarce.

The role of EVs in regeneration remains incompletely understood. Studies have shown that EVs harvested from stem cell cultures can enhance cell proliferation in both vertebrates and invertebrates (*26*–*28*). However, their specific roles in intercellular communication during adult regeneration in vivo have yet to be clearly demonstrated. Leveraging the remarkable regenerative abilities of the freshwater planarian *Schmidtea mediterranea* (*29*) and the recent findings that EVs promote stem cell proliferation in this organism (*26*), we investigated whether EVs are involved in regeneration. Planarians can regrow lost tissues within 7 days following amputation, a process involving rapid wound healing, the formation of a regeneration blastema, and subsequent differentiation into missing body parts. Although planarian stem cells are well recognized as key players in this process (*30*, *31*), our study reveals that biomolecules associated with the vesicular fraction of the cell secretome likely play a crucial role in planarian regeneration. We found that planarians modulate EV production under different growth conditions and uncovered a function of EVs as transporters for processed small interfering RNAs (siRNAs) during RNA interference (RNAi). RNAi has been a powerful tool for perturbing genes in planarians since 1999 and is a key technique in studying gene regulation during regeneration and homeostasis (*32*). Notably, transplantation of EVs isolated from RNAi-treated animals successfully replicated the RNAi defect in healthy specimens. These findings suggest that the vesicular secretome in planarians serves previously unrecognized but essential functions in cellular transport and regeneration, opening avenues for understanding the complex mechanisms underlying tissue regeneration.

## RESULTS

### Adult planarians and their stem cells secrete EVs

**Fig. 1.**
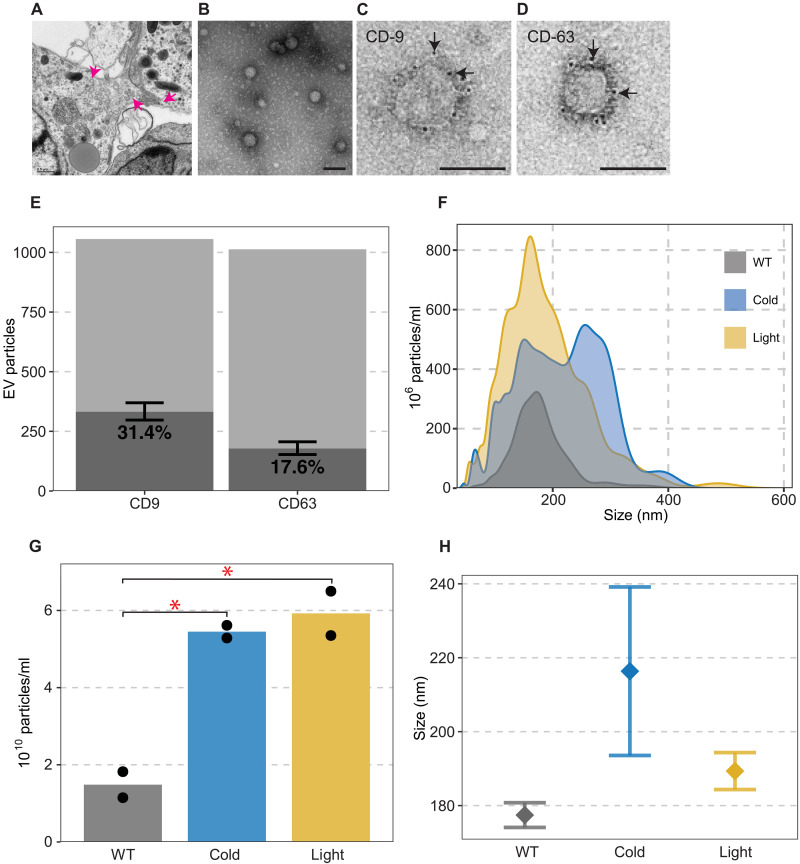
Presence of EVs in planarian cells. (**A**) TEM of 24-hour-cultured X1FS planarian stem cells shows the presence of endosomal vesicles (magenta arrows). (**B**) EVs isolated from cultured stem cells. Scale bar, 100 nm. (**C** and **D**) Characterization of planarian EVs using known EV markers CD-9 (C) and CD-63. (D) EVs were fixed onto EM grids and immunofluorescence stained for respective EV markers. (**E**) Percent of EVs labeled by CD9 (left) or CD63 (right) antibody as a percent of total EVs. Pale gray is total EVs, and dark gray is labeled EVs. Whiskers indicate 95% confidence interval of a one-sample Poisson rate test. CD-9 was detected more frequently than CD-63 (31.44% versus 17.57% of EVs). (**F** to **H**) Modulation of EV production in response to physical stimuli. Planarians were exposed to white light and cold (10°C); *n* = 200 animals per condition. NTA was performed to quantify EV particles. (F) Distribution of particle concentration versus size for each condition. (G) Total concentration of EV particles by condition. Black dots represent individual replicates. A two-sample *t* test was calculated between WT and the other two conditions. *P* values: cold, 0.023; light, 0.036. (H) Mean EV particle size by condition; diamonds indicate mean, and whiskers indicate individual values for two replicates.

To investigate whether EVs play a role in cell-cell communication in planarians, we first examined whether cultured planarian cells were capable of producing EVs. Cells enriched in stem cells were derived from tail fragments of planarians (*33*) and cultured within three-dimensional (3D) Matrigel domes for 24 hours before being fixed for transmission electron microscopy (TEM) analysis. Our observations revealed the presence of vesicles and MVBs near points of cell-cell contact (Fig. 1A, magenta arrows, and fig. S1A), suggesting that planarian stem cells may be capable of producing EVs. Following existing protocols (*34*), we isolated EVs from two conditions: planarian stem cell tissue culture and whole animal–rearing media. TEM confirmed the presence of EVs ranging from 50 to 200 nm in size (Fig. 1B and fig. S1B). In addition, immunogold electron microscopy (EM) labeling detected CD9 and CD63 in the purified particles (Fig. 1, C and D, arrows, and fig. S1, C to E), which are known to be enriched in EVs (*35*). Quantification of >1000 vesicles per antibody condition, we found that 31.44% of the particles were positive for CD9 and 17.57% for CD63 (Fig. 1E). Collectively, these data indicate that planarians secrete bilayered vesicles with sizes, morphology, and molecular components consistent with those of EVs.

Because EV secretion is known to be modulated by environmental stimuli (*36*), we examined whether the release of putative planarian EVs observed in our EM studies could be similarly influenced. To investigate the mechanism of EV release in planarians, we exposed these organisms to known stressors, specifically low temperatures and continuous light exposure (*37*). We used nanoparticle tracking analysis (NTA) to measure the release of EVs based on their light scattering and Brownian motion properties. The NTA revealed a significant increase in the production of small EVs (<200 nm) in animals subjected to either constant light or cold temperatures compared to those maintained under standard conditions (Fig. 1, F to H). Together, we conclude that planarians actively secrete vesicles indistinguishable from EVs described in other organisms and that the production of these particles is dynamically adjusted in response to environmental cues.

### Expression of EV biogenesis pathway genes is essential for planarian homeostasis

**Fig. 2.**
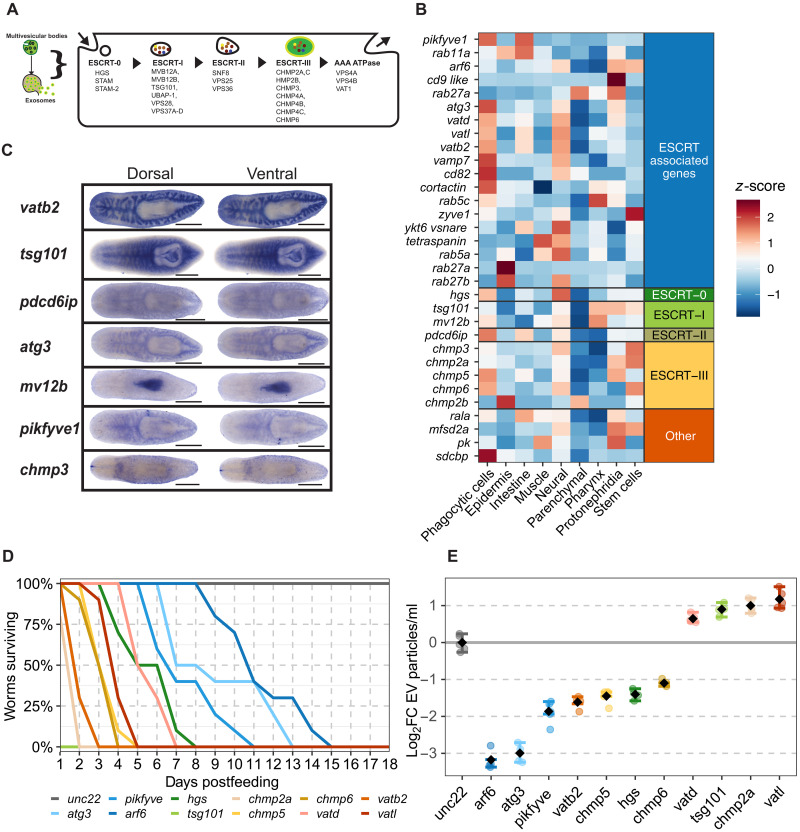
Genes involved in EV biogenesis. (**A**) Pictorial representation of known ESCRT complex participation in EV biogenesis. We found 32 genes associated with the ESCRT complex in planarians. (**B**) Expression of data ESCRT and ESCRT-associated genes in unirradiated regenerating planarian single-cell data from Benham-Pyle *et al.* (*39*), colored by the *z*-score of mean VST-normalized expression. (**C**) Whole-mount in situ hybridization reveals expression patterns of ESCRT complex and associated genes. Scale bar, 500 μm. (**D**) Survival of RNAi-treated animals (*n* = 10 animals per condition). Animals were fed three rounds of RNAi food spaced 4 days apart, and survival was tracked for 18dpf. (**E**) Modulation of EV production by members of the ESCRT complex and associated genes upon RNAi treatment (*n* = 200 animals per RNAi). NTA was performed to measure the production of EVs from different knockdown conditions. The data represent the mean log_2_FC of EV production in different RNAi conditions versus the *unc22(RNAi)* mean. Black diamonds indicate mean concentrations, whiskers indicate a 95% confidence interval for the mean, and dots represent technical replicates.

We identified and cloned 32 planarian homologs of genes known to be involved in the EV biogenesis pathway (*38*), which are primarily associated with ESCRT-dependent or ESCRT-independent pathways (Fig. 2A). To elucidate the expression patterns of these genes, we analyzed our lab-generated single-cell data (Fig. 2B) and performed mRNA in situ hybridization in homeostatic animals (Fig. 2C and fig. S2A). The mRNA expression profiles obtained from whole-mount in situ hybridization were consistent with our single-cell transcriptomics data (*39*). Our single-cell and in situ data reveal that phagocytic and neural cells have higher expression of ESCRT complex genes. To investigate the roles of these genes in planarian EV biogenesis, we used RNAi by feeding double-stranded RNA (dsRNA)–expressing bacteria to asexual planarians. Among the 32 genes, 11 exhibited phenotypes in intact animals, characterized by epithelial rupturing and head regression, indicative of tissue turnover defects (Fig. 2D and fig. S2B). The *Caenorhabditis elegans* gene *unc-22*, which is not expressed in planarians, served as a control for the knockdown experiment. Furthermore, we assessed the requirement of these biogenesis genes for EV secretion under normal conditions by performing NTA on RNAi animals of the 11 genes that caused homeostatic defects such as epithelial lesions and head regression (Fig. 2E and fig. S2C). These observations support an evolutionarily conserved role for ESCRT complexes in modulating EV production and highlight the critical role of EV biogenesis pathways in facilitating planarian regeneration and homeostasis.

### Proteomic and small RNA profiles reveal distinct molecules associated with planarian EVs

**Fig. 3.**
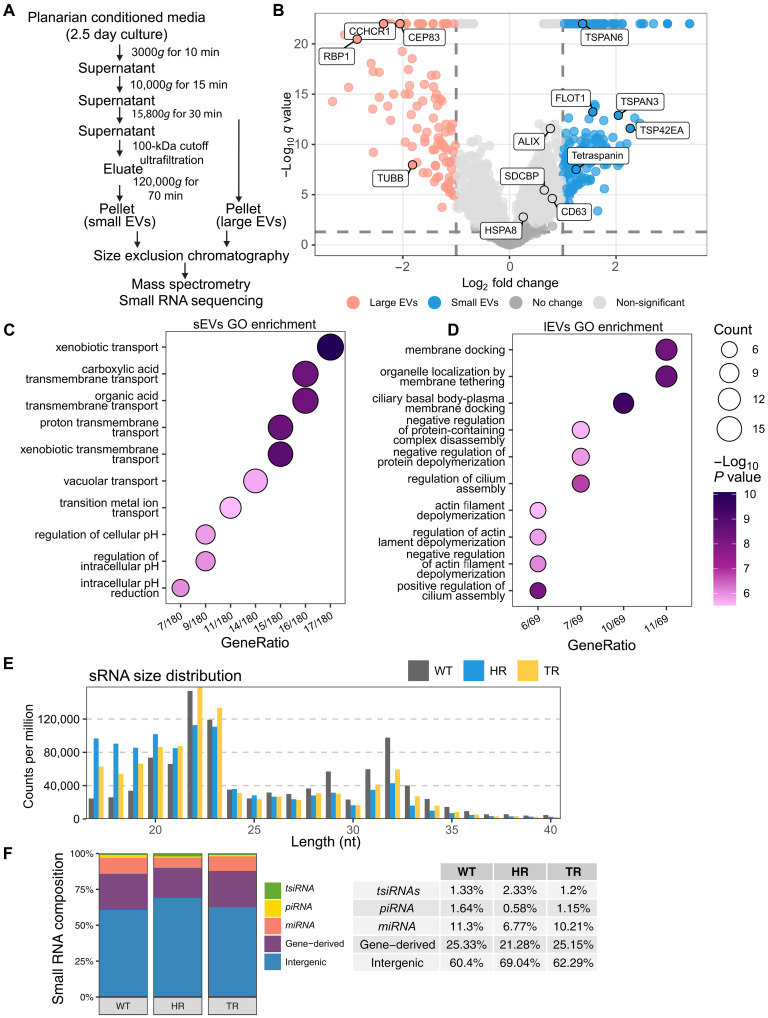
Proteomics and small RNA analysis of planarian EVs. (**A**) Methodology used to isolate small and large EVs from conditioned planarian media. (**B**) Volcano plot of the differential abundance of proteins in small versus large EVs isolated from homeostatic animals. The *x* axis is log_2_FC of abundance in small EVs versus large EVs. The *y* axis is significance calculated as −log_10_(*q* value). Blue dots represent proteins more abundant in small EVs, pink dots represent proteins more abundant in large EVs, and gray dots indicate nonsignificant proteins. (**C** and **D**) Enrichment of top GO terms (BP) for small and large EVs from WT animals. (**E**) Histogram representing the sRNA read length distribution in nucleotides, plotted as counts per million, from EVs isolated from WT (gray), HR (blue), and (yellow) animals. (**F**) Stacked bar plots of the fraction of different small RNA classes associated with EVs (piRNA, miRNA, gene-derived, intergenic, and tsiRNAs) in each experimental condition (WT, HR, and TR). Mean composition values are reported for each sRNA class. EVs were isolated from 800 WT, 800 head, and 800 tail fragments; *n* = 3.

Proteomics and small RNA analyses are often performed to better characterize and identify the information carried by EVs. We conducted extensive proteomics and small RNA analyses on the large and small EVs recovered from wild-type (WT) and regenerating animals (Fig. 3A). We operationally define “large EVs” as the pellet from 15,800*g* centrifugation and “small EVs” as those pelleted at 120,000*g*. Samples for proteomic analysis were prepared in triplicate using the “One-Tip” method (Materials and Methods) (*40*). Quantitative label-free proteomic analysis was performed using data-independent acquisition (DIA) methods with nanoflow liquid chromatography (LC) optimized for low-input samples. A total of 3312 proteins were detected across all samples, with most proteins present at similar levels in both EV classes. However, distinct subsets of proteins were enriched [log_2_ fold change (FC) > 1, 1% false discovery rate (FDR)] or uniquely identified in either large or small EVs.

In WT animals, proteomic analysis of small EVs showed enrichment for orthologs of canonical mammalian EV markers such as Flotillin 1 (FLOT1), TSPAN3, and several planarian-specific tetraspanins (*20*, *41*) (Fig. 3B). We also detected other EV markers such as ALIX (PDCD6IP), SDCBP (syndecan binding protein), CD63, etc. In contrast, proteins related to actin and cilia were enriched in the large EVs (table S1). This pattern was found to be generally the same in EVs isolated from regenerating animals with minor differences. For example, FLOT1 was less specific for small EVs during regeneration than in WT animals (fig. S3, A to C, and table S2). Gene Ontology (GO) analysis revealed enrichment of transport molecules in the small EVs and actin-related molecules in the large EVs (Fig. 3, C and D, and table S3), consistent with biogenic pathways described for small and large EVs in other systems (*20*). Overall, our quantitative proteomics analyses identified diverse sets of protein and putative markers enriched in small and large EVs, demonstrating that EV protein composition changes under different physiological conditions.

To test whether small noncoding RNAs (sRNAs) are associated with purified planarian EVs, we collected small EVs from WT, head-regenerating (HR), and tail-regenerating (TR) animals. Sequencing yielded 14,214,433 reads from WT, 19,535,879 from HR, and 16,661,896 from TR-derived EVs, respectively. Each condition was sequenced across three replicates. After removing noncanonical sRNA reads [i.e., reads too short to align <17–base pair (bp), >40-bp reads, nonadapter, ribosomal RNA (rRNA), and mitochondrial sequences], 8,494,455 reads from WT, 11,371,091 from HR-derived and 8,458,823 from TR-derived EVs aligned to the *S. mediterranea* genome (table S4). Consistent with findings from other organisms, the collected planarian EVs contained Piwi-interacting RNAs (piRNAs), miRNAs, and tRNA-derived sRNAs (tsiRNAs) (*42*, *43*). We used published data and databases to classify the miRNA, tsiRNAs, and piRNA populations (*44*–*46*). Analysis of sRNA sequences revealed that the majority mapped to the intergenic region of the genome (WT, 60.4%; HR, 69.04%; TR, 62.29%), followed by gene-derived sequences (including gene fragments and degradation products; WT, 25.33%; HR, 21.28%; TR, 25.15%) (Fig. 3E), whereas miRNAs accounted for ~6 to 11% across all three conditions, followed by lower proportions of piRNAs and tsiRNAs (Fig. 3F). Size distribution profiling showed a predominant peak at ~22 nucleotides, consistent with the size of mature miRNAs (fig. S3E). Additional peaks corresponding to other small RNA classes, including piRNAs [31 to 33 nucleotides (nt)] and tsiRNAs (17 to 40 nt), were also evident (Fig. 3F and fig. S3E). We observed a trend of sRNA sequence enrichment in TR-derived small EVs compared to HR-derived small EVs; for instance, *miR-124c*, a key regulator for neurogenesis (*47*), is enriched in TR-derived EVs (table S5). This comprehensive analysis not only defined the diverse RNA biotypes associated with planarian EVs but also revealed distinct sRNA profiles in EVs derived from HR- and TR-planarians, suggesting a potential role for EV-mediated sRNA transport in region-specific regeneration processes.

### Evidence for systemic, EV-mediated gene silencing in planarians

**Fig. 4.**
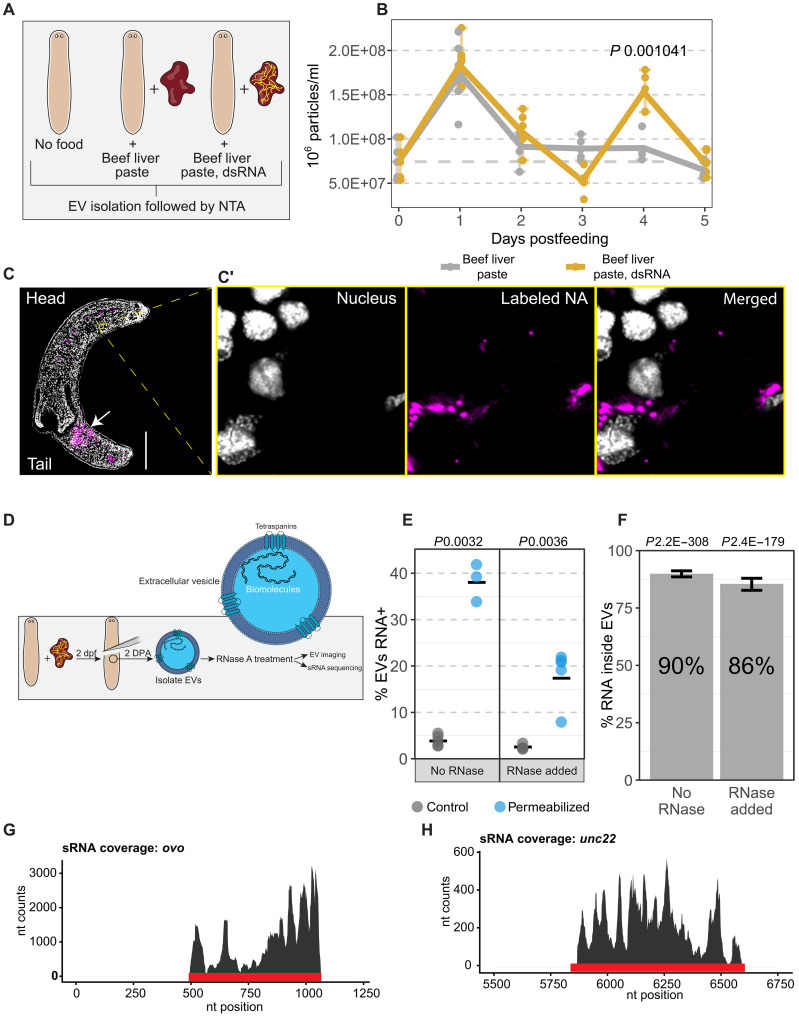
dsRNA-derived siRNAs are associated with small EVs. (**A**) Pictorial representation of different feeding regimes used for NTA. (**B**) NTA quantification of the small EV particle production of control (beef liver paste) and beef liver paste+*unc22* dsRNA–fed animals for 5dpf. Baseline “0dpf” values are from unfed animals. Points represent technical replicates, whiskers indicate a 95% confidence interval for the mean, and both the 95% confidence interval and *P* value are from a two-sample *t* test for fed versus dsRNA-fed animals at each time point. (**C** and **C′**) Digoxigenin-labeled *unc-22(dsRNA)* was injected post-pharyngeally in planarians. After 24 hours, they were fixed and coronally sectioned into 10 μm; magenta is digoxigenin-labeled dsRNA, and white is DAPI. Scale bar, 500 μm. (**D**) Workflow for single-particle EV imaging and small RNA sequencing after dsRNA feeding. A total of 800 animals were fed dsRNA for 2 hours. Animals were amputated 2dpf pre-pharyngeally, and EVs were isolated from the conditioned media 2 days post amputation. (**E**) dSTORM results showing the percent of EV particles positive for the dsRNA signal in control versus permeabilized samples in the absence (left) or presence (right) of RNAse. Points represent technical replicates. Bars indicate means for each condition, and *P* values represent a two-sided *t* test between control and permeabilized samples. (**F**) Nonpermeabilized EVs show a fraction of the dsRNA signal of their permeabilized counterparts in the absence (10%) or presence (14%) of RNase, indicating that most of the dsRNA signal is coming from RNAs packaged within EVs (90 and 86%, respectively). *P* values represent Poisson rate tests for dsRNA-negative versus dsRNA-positive EVs in each RNase condition, and black bars mark the 95% confidence interval. (**G** and **H**) dsRNA-derived siRNAs from EVs mapped back to their respective gene sequences. The red line represents the region where the dsRNA was designed. The black histogram represents the alignment of sRNAs in the gene sequence.

To explore the adaptive nature of planarian EVs in response to varying physiological conditions, we investigated how metabolic activity modulates small and large EV production. We fed planarians two types of food: beef liver paste and beef liver paste mixed with *unc-22(dsRNA)*. A group of unfed animals served as a baseline for EV production (Fig. 4A). Using NTA, we observed a drastic increase in small and large EV production at 1 day postfeeding (1dpf) under both fed conditions, with a significantly notable (*P* = 0.001) surge in dsRNA-fed animals at 4dpf with respect to small EVs (Fig. 4B). Unexpectedly, we did not find a similar trend with the large EVs (fig. S4A). This increase prompted us to test whether small EVs could serve as potential carriers for dsRNA-derived siRNAs, which are known for suppressing mRNA function.

Next, we injected digoxigenin-labeled *unc22(dsRNA)* into planarians and performed immunohistochemistry 1dpf. The labeled dsRNA dispersed throughout the planarian anatomy, with closer examination revealing its arrangement within the cell cytoplasm in a vesicular pattern, suggesting an association with endosomal-like structures that may ultimately become EVs (Fig. 4, C and C′). For detecting dsRNA within cells, we fed planarians custom-designed, digoxigenin-labeled dsRNA (scramble dsRNA) and isolated stem cell–like cells 2dpf. We used X1FS [X1 (irradiation sensitive) and FS (forward scattering)] cells, which are a heterogeneous population of neoblasts isolated without labeling using flow cytometry (*48*). We detected dsRNA signals within the cytoplasm of the cell marked by PIWI-1 antibody (fig. S4B). We then examined the EV composition of dsRNA-treated animals using two approaches. First, we used single-particle EV imaging to detect digoxigenin-labeled sRNAs in EVs. Second, we conducted small RNA sequencing to determine the presence of dsRNA-derived siRNAs associated with EVs (Fig. 4D).

For single-particle EV imaging, we fed animals with a scramble dsRNA and subjected the resulting EVs to single-particle EV imaging (Materials and Methods). EVs were labeled with a membrane lipid dye, fluorophore-conjugated antibodies targeting three tetraspanins (CD9, CD63, and CD81), and an anti-digoxigenin antibody specific to the labeled dsRNA or dsRNA-derived siRNA fragments. EVs were then immobilized on coverslips using phosphatidylserine (PS)–mediated capture for direct Stochastic Optical Reconstruction Microscopy (dSTORM). To test assay specificity, nonpermeabilized and permeabilized EVs were first tested for RNA content (Fig. 4E). Only 4% of nonpermeabilized EVs were positive, whereas 38% of permeabilized EVs were found to be positive (Fig. 4E and fig. S4C). These results indicate that little to no digoxigenin-labeled RNA is accessible to detection in the nonpermeabilized EVs, suggesting that most of the digoxigenin-labeled RNAs are likely found inside the vesicles. As an additional control, we subjected the nonpermeabilized and permeabilized EVs to RNase treatment to further test the distribution of RNAs associated with EVs. Nonpermeabilized EVs show a 34% reduction in signal when compared to no RNase treatment controls, whereas permeabilized EVs have a 54% reduction in signal. We conclude from these experiments that the RNA detected in EVs are likely predominantly carried as cargo. A Poisson rate test was performed to determine the relative percent of permeabilized EV RNA signal still present in nonpermeabilized samples (10% without RNase and 14% with RNase) (Fig. 4F). This portion of the signal is assumed to be from external RNA, whereas the remainder of the signal is from internal RNA. This indicates that most labeled RNAs were likely packaged within EVs (86 to 90%), rather than external (Fig. 4, E and F, and fig. S4C).

We then sought to determine the nature of the RNAs present in EVs. We fed in vitro–synthesized dsRNA mixed with beef liver paste targeting the *ovo* gene, which specifically depletes photoreceptor progenitor cells and used *unc-22* as control (*49*). EVs from regenerating animals were collected, treated with RNase, and subjected to sRNA sequencing (Fig. 4D). The results confirmed the presence of gene-specific siRNAs associated with EVs. Coverage plots and alignments generated using the Integrative Genomics Viewer (IGV) demonstrated precise mapping of dsRNA-derived siRNAs to the targeted gene sequence used as the dsRNA templates (Fig. 4, G and H). Together, these findings demonstrate that dsRNA-derived siRNAs are associated with EVs and suggest that EVs may facilitate their transport across various cell and tissue types.

### Ago-3 plays a role in the transport of dsRNA-derived siRNAs via EVs

**Fig. 5.**
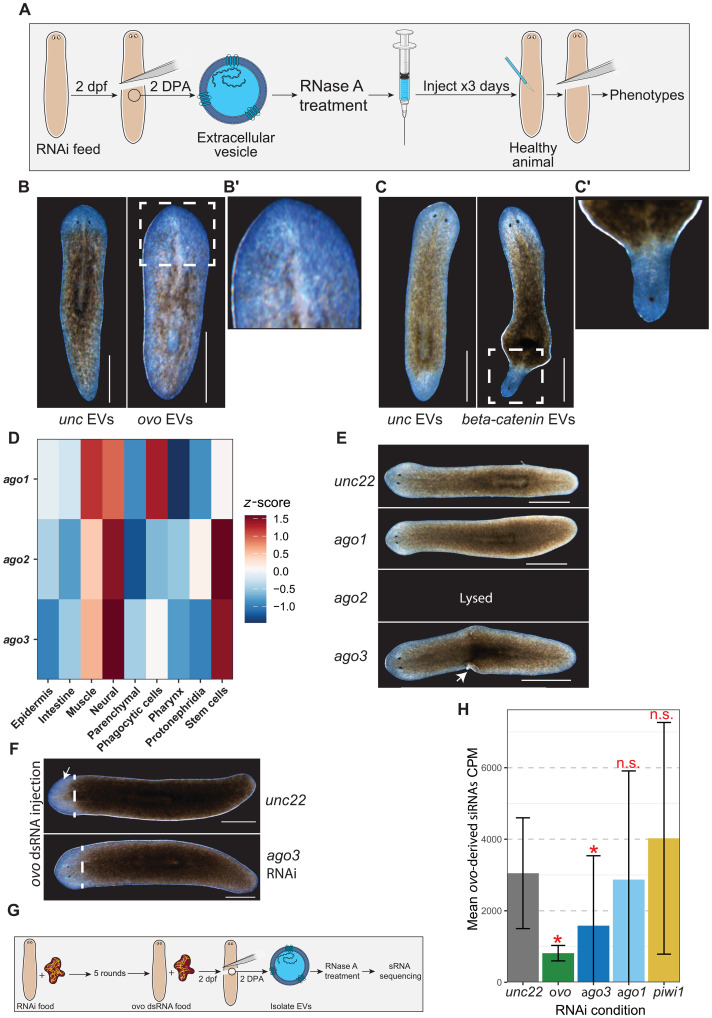
EVs harbor functionally active dsRNA-derived siRNA. (**A**) Workflow for isolating EVs after dsRNA feeding for transplantation. A total of 800 animals were fed dsRNA for 2 hours. Animals were amputated 2dpf post-pharyngeal. EVs were isolated from the conditioned media 2 DPA. Purified EVs were transplanted to healthy animals below the pharynx. (**B** to **C’**) EVs isolated from *ovo* and *beta-catenin* dsRNA-treated animals were transplanted into healthy animals. EVs isolated from *unc-22*–treated animals were used as a control for the study. After three rounds of EV transplantation, animals were amputated pre-pharyngeally. Animals transplanted with EVs isolated from *ovo* dsRNA-fed animals (*ovo* EVs, *n* = 4/5) were imaged 19 DPA, and *beta-catenin* dsRNA-fed animals (*beta-catenin* EVs, *n* = 10/12) were imaged on 10 DPA. Scale bars, 500 μm. (**D**) Heatmap showing the tissue-specific expression of *ago* gene across different cell types in nonirradiated regenerating planarian single-cell RNA sequencing data from Benham-Pyle *et al.* (*39*). (**E**) Representative images of *ago-1(RNAi), ago-2(RNAi)*, and *ago-3(RNAi)* animals. *n* = 10 animals per RNAi condition, Scale bars, 500 μm. (**F**) *ago-3(RNAi)* affects systemic RNAi. *ovo* dsRNA injection upon *ago-3(RNAi)* and *unc22(RNAi)*. When *unc22(RNAi)* produced no eye phenotype (8/9) after *ovo* dsRNA injection, *ago-3(RNAi)* failed to produce any eye phenotype (10/10). Images were taken 10 DPA. (**G**) Workflow to check dsRNA-derived siRNA association with EVs upon *ago* gene knockdown. (**H**) Small RNA sequencing quantification of *ovo* dsRNA-derived siRNAs associated with EVs after specific gene knockdowns. CPM, counts per million. Bars represent means, whiskers show 95% confidence intervals calculated as mean/−[1.96*SD/sqrt(*n*)] with *n* = 3, and *P* values are taken from a one-tailed *t* test against *unc-22(RNAi)*. *P* values: *ovo*, 0.011; *ago3*, 0.033. n.s., not significant.

The discovery that dsRNA-derived siRNAs are associated with EVs revealed a possible mechanism for effecting systemic RNAi in planarians. To investigate this phenomenon, we administered dsRNA targeting the planarian genes, *ovo* and *beta-catenin*. *ovo(RNAi)* results in the morphological absence of photoreceptor pigment cells (*49*), whereas *beta-catenin(RNAi)* disrupts anterior-posterior polarity and causes posterior amputation planes to regenerate a head instead of a tail (*50*, *51*). Planarians were amputated 2 days after three rounds of dsRNA feeding (Fig. 5A; Materials and Methods). EVs were collected from the planarian buffer 4dpf, treated with RNase A, and transplanted into healthy animals via microinjection in the posterior region just below the pharynx (Fig. 5A). Following 3 consecutive days of injections, we amputated the planarians post-pharyngeally and allowed them to regenerate. EVs from *unc-22* dsRNA-fed animals served as controls. The transplantation of EVs from *ovo* and *beta-catenin* dsRNA-fed animals resulted in gene-specific knockdown defects (Fig. 5, B, B′, C, and C′). Eighty percent (*n* = 4/5) of *ovo* EV-transplanted animals and 90% (*n* = 10/12) of *beta-catenin* EV-transplanted animals displayed the expected phenotypes. To further understand the important question of EV packaging of dsRNA-derived siRNAs mediating RNAi transmission, we performed transplantation experiments using permeabilized and nonpermeabilized EVs harvested from RNAi animals (Materials and Methods). Our results demonstrated that only nonpermeabilized EVs can elicit a strong RNAi phenotype (*beta catenin* EVs, 8/10; *ovo* EVs, 7/10), whereas permeabilized EVs failed to induce any observable phenotype (fig. S5A). These findings demonstrate the capacity of EVs to transport functionally active dsRNA-derived siRNAs across diverse tissues, providing strong evidence for their role in intercellular RNA communication.

The importance of RNA binding proteins (RBPs), such as ARGONAUTE, in sRNA sorting to EVs has been previously demonstrated in plants and parasitic nematodes (*52*–*54*). Therefore, we sought to determine whether the planarian *argonaute* genes *ago-1*, *ago-2*, and *ago-3* contribute to sRNA loading in EVs. Previous studies on *ago-2* in planarians have shown that disrupting this component of the RNA-induced silencing complex (RISC) leads to lethality. *ago-1* has failed to produce distinguishable defects after RNAi silencing, and *ago-3* remains understudied (*55*). Using available single-cell sequencing data (*39*), we attempted to define tissue-specific expression of *ago-1*, *ago-2*, and *ago-3* genes (Fig. 5D). The data indicate that *ago-2* and *ago-3* expression is enriched in stem cells whereas *ago-1* is not. We performed gene knockdown experiments to study the functions of *ago-1*, *ago-2*, and *ago-3* in planarians (Fig. 5E). All animals were fed every 3 days for a total of six times (Materials and Methods). *ago-1* (*n* = 10/10) and control-fed *unc-22(RNAi)* animals (*n* = 10/10) showed no defects 20 days after the last feeding, whereas *ago-2(RNAi)* animals (*n* = 10/10) lysed after two rounds of feeding. In contrast, *ago-3(RNAi)* animals survived but began to develop lesions soon after their last feed (*n* = 7/10) (Fig. 5E). To directly test *ago-3*’s role in systemic RNAi, we performed RNAi-mediated knockdown of *ago-3* followed by injection of gene-specific dsRNA (targeting *ovo*). In control animals, *ovo(RNAi)* leads to highly penetrant loss-of-eye phenotypes, consistent with effective systemic silencing (Fig. 5F). However, in *ago-3(RNAi)* animals, this phenotype was completely absent, despite receiving otherwise identical *ovo* dsRNA injections. This demonstrates that *ago-3* is required in recipients for gene-specific silencing by exogenous dsRNA.

Earlier, we showed that *ovo* siRNA molecules can be detected and quantified by sequencing EVs derived from *ovo* dsRNA-treated animals (Fig. 4, G and H). Therefore, we tested whether *ovo* siRNA numbers were altered in EVs obtained from *ago-1, ago-3*, and *piwi-1* RNAi backgrounds (Fig. 5F). After five rounds of RNAi feeding of *ago* and *piwi-1* genes, we introduced in vitro–synthesized *ovo* dsRNA into the treated animals. We isolated secreted EVs from the media of each of the RNAi-treated conditions and subjected them to sRNA sequencing. To minimize variability arising from differences in input material, we carefully quantified total EV RNA and initiated library preparation using matched input amounts for all samples and conditions. This approach allowed for the most direct comparison possible in the absence of spike-in controls (table S6). The experiments revealed a notable decrease in *ovo* dsRNA-derived siRNA levels in *ago-3(RNAi)* animals compared to *ago1* and *piwi1* knockdowns (Fig. 5G). As a control, we isolated sRNAs from whole animals to test whether the global processing of dsRNA into siRNAs was affected. Our data show no marked changes from the *ago-3(RNAi)* and *unc22* control (fig. S5C). We also examined the global depletion of distinct sRNAs in EVs following *ago-3(RNAi)*. Our analysis also reveals that the miRNA population was significantly reduced in sRNAs, apart from ovo dsRNA-derived siRNAs (fig. S5, D and E), whereas other populations remained unchanged. Moreover, we failed to detect siRNAs of nontarget genes in dsRNA-treated animals (fig. S5F). In conclusion, our study provides evidence that EVs transport functionally active dsRNA-derived siRNAs across tissues, with *ago-3* playing a crucial role in the functional association of active dsRNA-derived siRNAs with EVs.

### Cultured planarian cells uptake purified EVs

**Fig. 6.**
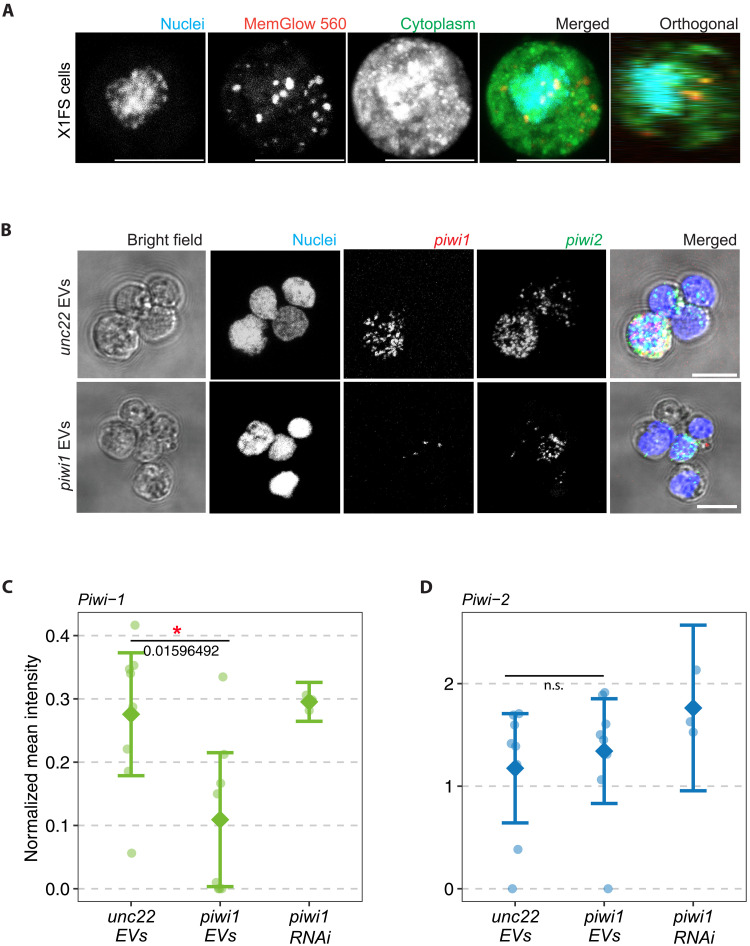
Planarian X1FS stem cells uptake labeled EVs. (**A**) Membrane-labeled planarian EVs can fuse with planarian X1FS cells. X1FS cells were labeled with CellTracker Green live stain. EVs were labeled with an EV membrane stain before addition to the culture media, and nuclei were costained with Hoechst 33342. Scale bars, 10 μm. (**B**) *piwi-1* knockdown on X1FS cells using EVs harvested from *piwi-1(RNAi)* animals. EVs were isolated from 500 animals after two rounds of RNAi feeding. Purified EVs were added to X1FS cells (150,000 cells per well) and cultured at 20°C, 5% CO_2_ for 3 days. The cells were fixed using 4% PFA, and HCR was performed. X1FS cells were stained for *piwi-1* and *piwi-2* using HCR probes. *piwi-2* HCR was performed to show the specificity of EV-mediated RNAi. (**C** and **D**) Box plot represents the mean intensity of one field of view of cells from the HCR in situ; points are technical replicates, whiskers indicate a 95% confidence interval for the mean derived from a one-sample *t* test, and *P* values come from a two-sample *t* test against worms incubated with EVs from *unc22(RNAi)* worms. X1FS cells from *piwi-1(RNAi)* animals were used as a negative control. *, significant; n.s., not significant.

We sought to determine whether purified EVs could be taken up by cultured planarian cells. For our tests, we used sorted X1FS from whole animal macerate. X1FS cells were incubated with membrane-stained EVs for 16 hours and subjected to confocal microscopy. We used spectral imaging and linear unmixing to eliminate background noise and autofluorescence. The results show intracellular EV localization, with orthogonal images confirming labeled EV uptake (Fig. 6A). To test whether EVs taken up by cells could deliver their cargo, we purified EVs from *piwi-1(RNAi)*–treated animals. We selected this gene because planarian stem cells are enriched in *piwi1* expression, yet knocking down this transcript does not produce detectable phenotypes (*56*) and would not be expected to alter the viability of X1FS cultured cells. We treated freshly sorted X1FS cells with EVs isolated from *piwi-1(RNAi)* animals (Fig. 6B). X1FS cells were maintained in culture for 4 days, and fresh EVs were added every 2 days. EVs from *unc-22*–treated animals served as controls. After 4 days, the cells were fixed and stained for *piwi-1* and *piwi-2* as control for RNAi specificity using HCRv3. Quantification of *piwi-1* signal mean intensity revealed a significant decrease (*P* = 0.015) in *piwi-1* transcripts in cells treated with *piwi-1(RNAi)*–derived EVs (Fig. 6, B and C). We also observed a subtle difference (*P =* 0.6) in *piwi-2* transcripts in these cells (Fig. 6D). As an additional control, X1FS cells were isolated from *piwi1(RNAi)* animals, showing a similar normalized mean intensity signal to the cells cultured with EVs (Fig. 6, C and D). These findings highlight the potential of EVs as an intercellular communication mechanism in planarian biology, demonstrating their ability to effectively transport and deliver functional RNA molecules to target cells.

## DISCUSSION

**Fig. 7.**
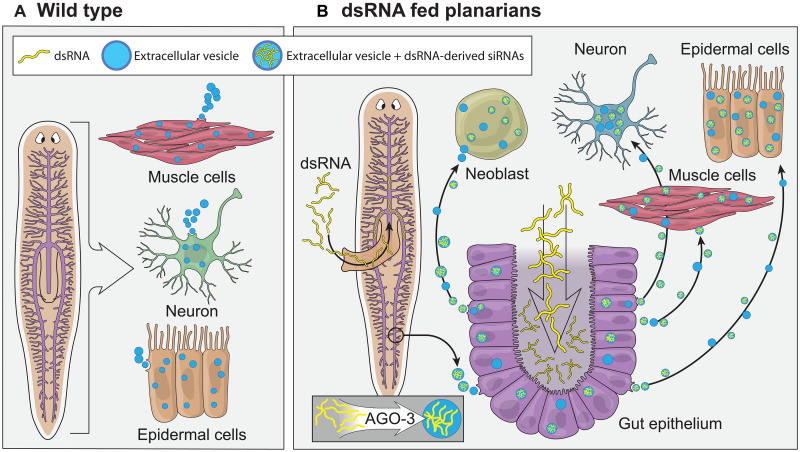
Planarian cells make EVs. (**A**) Planarian cells produce EVs. (**B**) Upon dsRNA ingestion (yellow), dsRNA is broken down to siRNAs (small yellow fragments). With the help of AGO-3, EVs transport dsRNA-derived siRNAs from one cell type to another, aiding systematic RNAi.

Our study provides evidence for the functional importance of EVs in planarian biology, particularly in the context of regeneration and intercellular communication. Our data also revealed that planarian EV production is dynamically regulated in response to environmental stressors, suggesting a role for these vesicles in adaptive responses. In addition, we have shown that planarians, like other organisms, secrete EVs that conform to established size and morphological criteria (*57*) (Fig. 7A). The presence of CD9 and CD63 markers on these vesicles further supports their identity as EVs, aligning with observations in other species (*35*, *58*). However, future studies involving RNAi-mediated knockdown of planarian CD9/CD63 orthologs followed by immunoblotting and immunofluorescence will be required to further confirm these findings.

### EV biogenesis genes are evolutionarily conserved in planarians

The identification and characterization of 32 planarian homologs of genes involved in EV production in other species (including 26 mammalian homologs) indicate that EV biogenesis appears to be evolutionarily conserved in planarians. These genes include ESCRT proteins, which have been shown to play key roles in EV biogenesis across a wide range of organisms, from bacteria to humans (*38*, *59*, *60*). Knockdown experiments using RNAi demonstrated that perturbing the expression of the 32 homologs identified genes modulated EV production. Of the 32 homologs identified, only 11 yielded detectable phenotypes (Fig. 2D and fig. S2B). For the remaining genes, we failed to observe RNAi phenotypes, suggesting either redundancy or limitations in RNAi efficacy. NTA confirmed altered EV production under these 11 RNAi-silenced conditions, likely due to inefficient vesicle production or tissue damage (Fig. 2E). The high degree of conservation in EV biogenesis mechanisms underscores the critical role of EVs in animals in general and in planarian tissue maintenance and regeneration in particular. Our findings reveal biological contexts for investigating the connection between EV production and tissue homeostasis, as well as regenerative processes. Although the mechanisms of EV biogenesis and secretion are not yet fully understood, the experimental accessibility of EV biogenesis and function in planarians provides opportunities to understand EV-related processes across different species. This could potentially inform future studies on tissue regeneration and homeostasis in organisms like vertebrates.

### Distinct biomolecules associate with small and large EVs

Our comprehensive proteomic and small RNA analyses of planarian EVs revealed a complex and diverse collection of biomolecules. The study identified 3312 proteins across samples, such as the EV biogenesis protein CHMP2A. We also uncovered distinct profiles for large and small EVs. For example, small EVs were enriched in transport molecules and canonical EV markers like tetraspanins, and flotillin1, whereas large EVs had more cytoskeletal (e.g., actin and tubulin) modifiers (*17*, *20*). Moreover, the presence of various RNA species, including miRNAs, tsiRNAs, and piRNAs, in planarian EVs is particularly intriguing. Future experiments incorporating permeabilization will provide better resolution of sRNAs packaged inside the EVs. This remarkable diversity and specificity of biomolecules found associated with planarian EVs suggest a sophisticated intercellular communication system that may be crucial in orchestrating complex biological processes such as regeneration and physiological turnover.

The distinct protein profiles observed in large and small EVs, along with the differential enrichment of RNA species in EVs derived from regenerating head and tail tissues, point to a highly regulated and context-dependent mechanism of EV biogenesis and specialization. This tailored approach to EV content based on physiological state implies that planarians may have a highly regulated system for transmitting molecular information between cells. This type of regulation may potentially allow precise coordination of cellular behaviors during tissue regeneration and physiological conditions. Such a system could enable dissemination of regenerative signals throughout the organism and may be, in part, responsible for the remarkable regenerative capabilities observed in planarians.

### EVs as mediators of RNAi

One of the most notable findings of this study is the role of EVs in gene silencing. In other organisms such as *C. elegans*, EVs have been shown to serve as a potential carriers for endogenous RNAs and to carry out interspecies RNA communication (*61*). RNAi has been used in planarians for more than two decades to silence gene expression (*32*, *62*, *63*), but little is known about the way in which RNAi works systemically in these organisms. We observed a significant increase in EV production following dsRNA ingestion, and high-throughput small RNA sequencing of EVs isolated from RNAi-fed animals revealed the presence of dsRNA-derived siRNAs (Fig. 4, C and C′). When digoxigenin-labeled dsRNA was fed to planarians, single-particle EV imaging showed that most digoxigenin-labeled RNAs appear to reside inside the vesicles (Fig. 4, E and F). Permeabilized EVs showed a higher percentage of RNA positivity compared to nonpermeabilized ones, indicating that most RNAs are encapsulated within EVs. Moreover, permeabilized EVs were less affected by RNase treatment, further supporting that RNAs are likely and predominantly carried as cargo inside EVs. Transplantation of EVs from dsRNA-fed planarians into healthy animals elicited RNAi responses, leading to specific gene knockdowns (Fig. 5, B, B′, C, and C′). The uptake of EVs by planarian cells was confirmed using confocal microscopy, showing internalization and gene knockdown effects in cells treated with *piwi-1(RNAi)*–derived EVs (Fig. 6, A to C). However, a direct comparison of uptake and RNAi efficiency between exogenous dsRNA and RNAi-derived EVs would display the potential ability of EV-mediated RNA transfer. Overall, our study suggests that EVs can serve as carriers for dsRNA-derived siRNAs. Thus, the ability of EVs to deliver functional RNAi molecules across tissues represents a previously undiscovered mechanism for systemic gene silencing in planarians. This discovery not only sheds light on the efficiency of RNAi in these organisms but also suggests potential applications for targeted gene manipulation in regenerative studies.

### Role of Argonaute proteins in EV-mediated siRNA transport

In planarians, the function of *ago-1* remains uncertain, whereas *ago-2* is essential for viability (*64*) and presumably for miRNA-based RISC. Our work uncovers a role for planarian *ago-3* in modulating siRNA packaging into EVs (Fig. 7B). Knockdown of *ago-3* resulted in a significantly lowered abundance of dsRNA-derived siRNAs associated with EVs (Fig. 5G). These results suggest a role for AGO-3 in the association of siRNAs with EVs. Planarians have three AGO proteins: AGO-1, AGO-2, and AGO-3. AGO-2 is associated with miRNA biogenesis, and *ago-2(RNAi)* is lethal to planarians. Knockdown of *ago-1* fails to produce any noticeable phenotypes even after extensive feeding (*55*), whereas *ago-3(RNAi)* results in lesions in the animals. Studies on plant EVs have shown AGO-1 and other RBPs to play a crucial role in associating sRNAs with EVs (*52*). An extracellular form of AGO (WAGO) was also shown to be involved in modulating the siRNA composition of EVs in parasitic nematodes (*54*). In fission yeast, AGO-1, in association with CID14 and CID16, maintains RNA surveillance and prevents uncontrolled endogenous gene silencing (*65*). Identifying planarian *ago-3* as an important player in the association of siRNAs with EVs provides important mechanistic insights into this process. Upon *ago-3(RNAi)*, no disruption of the global small RNA processing was observed, but we detected a noticeable decrease in the association of dsRNA-derived siRNA with EVs (Fig. 5G and fig. S5C). Although *ago-3* is genetically required for the EV-mediated silencing effect, proteomic analyses failed to detect AGO-3 in EVs, suggesting that AGO-3’s critical function may precede EV export, potentially by initiating or facilitating the generation, selection, or stable loading of siRNA cargo into vesicles for subsequent transfer. The differential roles of *ago-2* and *ago-3* in planarian biology, combined with EV-mediated siRNA transport, highlight the complexity of RNA regulatory mechanisms in these organisms. Further studies on the interplay between Argonaute proteins and EV biogenesis genes (e.g., ESCRT components) using alternative strategies such as tissue-specific, partial, or temporally controlled knockdown are the critical next steps to elucidate the function of RBPs and EVs. These studies could reveal new aspects of gene regulation in homeostasis and regeneration.

Extensive research on EVs across diverse biological systems has revealed their multifaceted roles in intercellular communication and physiological processes. From cancer progression (*22*) to microbial interactions (*59*, *60*), EVs have emerged as crucial mediators of information transfer capable of reprogramming cellular states and influencing cellular microenvironments. The discovery of EV-mediated siRNA transport and the involvement of *ago-3* in planarians sheds light on gene regulation mechanisms in these organisms and opens avenues for exploring EV functions across species. As the intricacies of EV biogenesis, cargo selection, and fate in living systems are resolved, it may be possible to harness the potential of EVs as highly adaptable tools for manipulating biological systems, particularly in regenerative biology and medicine. The ability to manipulate EV content and cellular targets presents exciting possibilities for developing strategies to reshape normal and abnormal biological processes. The proposed working model suggests that all cells in planaria are capable of making EVs, but this has yet to be experimentally determined. Future research focusing on EV-mediated communication in natural habitats and across species boundaries promises to deepen our understanding of ecological interactions and evolutionary processes, potentially revolutionizing our approach to studying and manipulating biological systems.

## MATERIALS AND METHODS

### Planarian husbandry

Asexual *S. mediterranea* (clonal line CIW4) animals were maintained at 20°C in 1x Montjuic water in Ziploc containers (*32*). Animals sized 4 to 6 mm were fed every other week with beef liver paste. Water was changed every second day after feeding. Animals were starved for a week before performing experiments.

### EV isolation and concentration

Five hundred asexual planarians, 4 to 5 mm long, were cultured in Ziploc containers in 0.1 μ filtered Montjuic water without antibiotics. The animals were cultured at 20°C for 2.5 days. Conditioned Montjuic water was collected and centrifuged at 3000*g* for 10 min. The supernatant was collected and centrifuged at 10,000*g* for 15 min. The supernatant was transferred to a new tube and spun at 15,800*g* for 30 min to obtain a pellet of large EVs. The supernatant was collected and transferred to a 100-kDa cutoff Amicon filter tube. The tube was spun at 3000*g* for 15 min to concentrate the supernatant to 4 ml as per the manufacturer’s instruction. The concentrate was then transferred to a 4-ml ultracentrifuge tube and subjected to 120,000*g* for 90 min to obtain a pellet of small EVs.

### EV isolation by Izon qEV columns

EVs were isolated according to the column manufacturer’s instructions. Briefly, column performance was monitored by the timing of the flow-through of wash buffer fully through the column. The small EV pellet from ultracentrifugation was resuspended in 400 μl of 1X phosphate-buffered saline (PBS). This was loaded into the qEV column, followed by adding 200 μl of PBS aliquots until the collection was finished. As recommended, we avoided the first 1 ml of fluid that elutes from the qEV column. We then collected the subsequent 600-μl EV fractions. The EV fractions were then concentrated by ultracentrifugation (110,000*g* for 1 hour) and resuspended in 30 μl of PBS.

### Nanoparticle tracking analysis

As previously described, NTA was performed to determine particle size and concentration [Hung *et al.* (*66*)]. Briefly, EV fractions were diluted in PBS from 1:50 to 1:1000 for different samples and analyzed using the NanoSight LM10 instrument (Nanosight, Salisbury, UK). Samples were captured in four 60-s videos. Data were collected at 25°C, with the camera level at 11. Data analysis was performed using NTA version 2.3 (*66*).

### EV negative staining and immunoelectron microscopy

Five microliters of the diluted EV pellet was placed on an EM grid and incubated for 2 min. After removing the excess solution, the grid was stained with a drop of 2% uranyl acetate (UA) for 2 min. Excess UA was wicked away using filter paper, and the grid was left to air dry. The samples were then examined and imaged with a Thermo Fisher Scientific Talos 200-kV transmission electron microscope. For immunoelectron microscopy, fixed EVs were blocked using 1% bovine serum albumin (BSA), followed by 1 hour of primary antibody incubation. Excess antibodies were washed away using blocking solution. Primary antibodies were detected by 6-nm Colloidal Gold Donkey Anti-mouse IgG. Purified Mouse Anti-Human CD9 primary antibodies were purchased from BD Pharmingen (555370), and mouse Anti-Human CD63 antibodies were purchased from Invitrogen (10628D).

### MemGlow staining for EV uptake assay

Fluorescence-activated cell sorting (FACS) was performed as described by Wagner *et al.* (*48*). A total of 5 × 10^5^ X1(FS) cells were sorted and plated into a PerkinElmer 384-well glass-bottom dish. Postsorting, the cells were stained by CellTracker Green per the manufacturer’s instructions. Purified EVs were stained with 200 mM MemGlow 560 for 1 hour at room temperature (RT). To remove excess stain, the labeling mixture was subjected to 100-kDa ultrafiltration. The concentrate was diluted again by 1x PBS and ultracentrifuged at 120,000*g* for 1 hour. The labeled EVs (3.4 μg/ml) were resuspended in 100 μl of 1x PBS. One microliter of labeled EVs was added to sorted X1(FS) cells and imaged overnight on an LSM 980 confocal microscope using a 63x oil immersion objective.

### Gene cloning

Cloning to produce constructs for RNAi and in situ hybridization was performed as described previously [Benham-Pyle *et al.* (*39*)]. Genes of interest were targeted by primers generated by Primer 3 (https://primer3.ut.ee) against the Sánchez Alvarado Lab transcriptome. These primers were designed to amplify fragments 40 to 600 bp in size from planarian cDNA, and the primers were modified for Gibson Assembly by the addition of 5′ sequences homologous to the pPR-T4P RNAi vector. Purified polymerase chain reaction (PCR) fragments were assembled with prepared vector and transformed directly into the *Escherichia coli* strain HT115, which facilitates efficient expression of dsRNA. Clones were validated by Sanger sequencing before use.

### dsRNA synthesis, labeling, and imaging

For dsRNA synthesis, gene of interest was cloned into a vector with T7 bacteriophage promoter sequence overhangs. The MEGAscript RNAi Kit was used to prepare dsRNA for feeding as per the manufacturer’s description. For dsRNA labeling with digoxigenin, the Digoxigenin-11-UTP kit was purchased from Sigma-Aldrich and mixed with the UTP from MEGAscript RNAi Kit to match the same concentration as other nucleosides. dsRNA synthesis and labeling were performed as per the manufacturer’s description. For dsRNA detection in whole animals, planarians were injected with digoxigenin-labeled dsRNA and, after 24 hours, fixed and coronally sectioned into 10-μm slices. Anti-digoxigenin antibody procured from Roche was used for detection.

### RNAi feeding

Unless otherwise specified, worms were fed dsRNA-expressing bacteria mixed with beef liver paste every 3 days for three RNAi feedings. For functional knockdown of *ago* genes and *piwi1*, planarians were fed every 3 days for six RNAi feedings. After respective RNAi feedings, animals were screened for phenotypes. dsRNA feeding was performed as mentioned in (*62*). Feeding digoxigenin-labeled dsRNA and unlabeled dsRNA yielded similar RNAi effects.

### Scrambled dsRNA sequence

A 377-bp scrambled sequence was designed, in vitro synthesized, and cloned into a pUDCIT vector from IDT. RNAi was performed as mentioned in (*62*). Briefly, 1000 planarians were fed with a liver solution containing digoxigenin-labeled dsRNA (20 μg). Small EVs were isolated from these animals, as mentioned previously.

Scramble sequence: GTTTGTTTTGTTTATTTTATGTGTCCGCAGTTTATCGGCACATTGCGTTTTTGACCCGAATTATTCTTTGGGTAACTTTGCACGTCTTCTAAAATTTCGACATGACCTTAGTATGGCCGTCTTGACGGTTATATAGGGCTAGTTCCACCATCGGTCGCTTCGTGGCGTCGTATAATGACAGATCTCACCAGTCTATCTATAACTAAGTCCGTATGCGTGAGTGGCATTGTCCGCTTGTACTCCTAGTTTTTCGATATTCTGTGTCTACATGGGACTTCTCCGTGCTTCCTTTTTTCGTGTTAGGTTTGACTGTCTCTATGGTCGTAATCCTATGGTAACGTTATGGCTGCATCTGTTTTTTTTCACTTGCCTCGTTTACTCAATCTGGTCCCTAGTGGTAGTAATACTAGCCATTGAAGCCACGACCACCTATTTTGGTTGCCGGGCTTTTTTTCCCTAAATCTTACTCAAGCGCCTTTTTTTAAATTTTATTGCTTATT.

### dSTORM imaging

Small EV samples were prepared for dSTORM microscopy according to the ONI EV Profiler 2 Kit. Briefly, 10^10^ to 10^11^ small EVs were captured on the chips via PS capture molecules. After fixation with 4% paraformaldehyde (PFA; reagent provided in the kit), small EVs were incubated for 1 hour at RT with fluorescently labeled PanEV membrane stain (488 nm), anti-CD63/CD81/CD9 (561 nm), and anti-digoxigenin (1.89 μg/ml, 640 nm). Permeabilization (reagent provided in the kit) and RNase treatment (1 mg/ml for 30 min, RT) were performed after fixation before antibody incubation. Antibodies and stains were prepared according to the ONI EV Profiler 2 Kit protocol, and samples were washed before postfixation with 4% PFA and then mounted using dSTORM imaging buffer. Images were acquired on the ONI Nanoimager using three laser channels (488, 561, and 640 nm) with a 100× magnification, 1.45–numerical aperture (NA) objective lens (field of view size = 50 μm by 80 μm) and analyzed using CODI software.

### Poisson rate test calculation

A Poisson rate test was used to calculate the dsRNA signal in nonpermeabilized EVs as a percent of the dsRNA signal in permeabilized EVs. This “nonpermeabilized” signal is the estimation for the proportion of dsRNA outside the EVs, whereas the remainder (100% minus nonpermeabilized %) is dsRNA encapsulated as cargo within EVs. Ten percent of the total (permeabilized) signal is present without permeabilization, so about 10% of the RNA+ EVs have RNA accessible from the exterior. Then, we use the value 1 − 0.1 = 0.90(100) to estimate that 90% of RNA is inside EVs. We use the same calculation on the 95% confidence interval from the Poisson test for the confidence interval on the plot.

### EV permeabilization and injection

EVs isolated from dsRNA-fed animals (*ovo* EVs, 1.07 μg/μl; *beta catenin* EVs, 0.9 μg/μl) and transplanted post-pharyngeally using a Drummond nanoject II injector. The EV pellet was resuspended in 50 μl of 1X PBS. EVs were transplanted (207 nl) every day for 3 days, which was equivalent to the yield from ~5 to 10 planarians, and amputated above the pharynx on the fourth day. From small RNA sequencing analysis, we determined that (~0.3%) of total EV small RNA in WT is specific to *ovo* siRNAs (Fig. 5G). On the basis of RNA yield per planarian and injection volume, we estimate that each EV injection delivers ~15 to 18 ng of total EV RNA, corresponding to ~27.9 to 33.5 pg of *ovo* siRNA. By contrast, direct injection of *ovo* dsRNA delivers orders of magnitude more template RNA, resulting in a substantially higher effective siRNA dose after processing. For the EV permeabilization followed by transplantation experiment, EVs were treated with Triton X-100 (0.1%) for 5 min at RT and repurified using ultracentrifugation.

### In situ hybridization

Planarians were fixed using a carboxylic acid protocol for whole-mount in situ hybridization (*67*). Samples were cleared overnight using SCALE A2 solution and mounted on glass slides the following day.

### Fluorescent in situ hybridization and analysis

Sorted X1FS cells were plated in a glass-bottom 96-well plate and allowed to rest for 4 days. One microgram of freshly isolated EVs was added to the dishes (piwi1 EVs at 0.51 μg/μl and unc22 EVs at 0.77 μg/μl). EVs were supplemented to the media every second day. On the fourth day, cells were fixed with 80% methanol and stored overnight at 4°C. Hybridization chain reaction (HCR) was performed as described in Molecular Instruments protocols for generic cells on a slide using probes *piwi-1* (GenBank ID DQ186985.1) and *piwi-2* (wormbase ID h1SMcT0019482.1) in the B1 and B2 amplifiers, respectively. The B1-AlexaFluor647 and B2-AlexaFluor488 hairpins were used for gene detection, and lastly, 4′,6-diamidino-2-phenylindole (DAPI; 0.2 mg/ml) solution was stained for all nuclei. Cells were imaged in PBS with a Zeiss 980 confocal microscope with a 63x, 1.4-NA objective using optimal settings for the dyes chosen. At least three regions were imaged per condition, including a negative HCR control. Image analysis was completed in Python 3.9. The cellpose cyto model (Pachitariu and Stringer, 2022) was used to segment maximum projections of nuclei of the X1FS cells and then expanded by 3 pixels to find the mean intensity of the *piwi-1* and *piwi-2* channels in the cells. After intensity normalization, a common threshold was chosen to determine which cells were positive for gene expression.

### One-Tip proteomics sample preparation

Lysis and digestion of EVs for proteomics analysis was carried out using the EvoTip “One-Tip” sample protocol as published [Ye *et al.* (*40*)]. Briefly, EvoTips were prepared according to vendor instructions, including rinsing with 20 μl of Solvent B (0.1% formic acid in acetonitrile), conditioning with isopropanol, and equilibration with 20 μl of Solvent A (0.1% formic acid in water). Next, 5 μl of 2x lysis and digestion buffer [0.2% *n*-dodecyl-β-d-maltoside (DDM), 100 mM triethyl ammonium bicaarbonate (TEAB), trypsin (20 ng/μl), and Lys-C (10 ng/μl)] were manually added to the prepared EvoTip. EVs isolated as described above were resuspended in PBS, and 5 μl (equivalent to EVs from 15 animals) of EVs/PBS was added to the lysis and digestion buffer. Samples were briefly centrifuged (10 s, 50*g*) and incubated at 37°C in an EvoTip box with Solvent A in the bottom. After 4 hours of digestion, 50 μl of Solvent A was added to each tip and centrifuged for 1 min at 800*g*, followed by two washes with 100 μl of Solvent A. Tips were then filled with 200 μl of Solvent A and stored at 4°C until acquisition.

### Liquid chromatography–tandem mass spectrometry

Samples (*n* = 3, technical replicates) were analyzed by liquid chromatography–mass spectrometry (LC-MS/MS) on a Bruker timsTOF flex MS coupled to an Evosep One HPLC system. LC was performed using the standard Evosep Whisper 40 SPD (31-min gradient) LC methods and a commercial analytical column (ionopticks Aurora Elite CSI, 15 cm by 75 μm) with the Bruker CaptiveSpray source. MS/MS data were acquired using timsControl (v.5.0) using default DIA with parallel accumulation and serial fragmentation (“DIA-PASEF–long gradient”) methods. Briefly, parameters used included positive ion polarity, trapped ion mobility spectrometry (TIMS) set to “On,” 100-ms ramp and accumulation time, and capillary voltage at 1500 V. DIA-PASEF windows covered a scan range of 400 to 1201.0 *m/z* (mass/charge ratio), and a mobility range from 0.6 to 1.60 1/*K*_0_, with a 1.8-s cycle time, and collision energy ramping from 20 eV at 1/*K*_0_ = 0.6 to 59 eV at 1/*K*_0_ = 1.60. Windows were 26-Da wide with 1-Da overlap, with 32 steps per cycle.

Raw data (Bruker.d) were converted to HTRMS format using the HTRMS Converter (v19.0, Biognosys) and searched using Spectronaut (v.19) using directDIA+ (deep) methods against a FASTA database containing 36,755 proteins including 426 common contaminants. Relevant parameters for the Pulsar search settings included specification of Trypsin/P + LysC/P for enzymes, acetyl (protein N-term) and oxidation (M) as variable modifications, 0.01 FDR control at the PSM, and Peptide and Protein level for identification. DIA analysis settings included 0.01 cutoffs for Precursor and Protein (both Experiment and Run) *Q* value and PEP, with exclusion of single hit proteins. Quantification was performed at the MS2 level using peak area. Spectronaut search results were exported at the peptide level using the report format for MS-DAP (*68*). Normalization and differential expression analysis were performed using the msdap R package (v1.0.8) in R (v4.4.1). Peptides were filtered to require identification and quantification in all three samples per group, with a minimum of two peptides per protein. Data were normalized using a combination of variance stabilizing normalization (VSN) with protein-level mode-between [norm_algorithm = c(“vsn”, “modebetween_protein”)], and differential expression was performed using DEqMS withing MS-DAP, with by-contrast filtering enabled (filter_by_contrast = TRUE). All other parameters used their default settings. For visualizations and GO analyses, significant hits were defined as proteins with absolute log_2_FC values greater than 1.0 (at 1% FDR) from DEqMS results or proteins identified from MS-DAP differential detection (DD) results with an absolute *z*-score greater than 4.0. For volcano plot visualizations, DD proteins used the log_2_FC values calculated by MS-DAP, and *q* values were set to the minimum *q* value for DE proteins in the plot. The mass spectrometry dataset generated for this study is available from the MassIVE data repository using the identifier MSV000097862.

### Small RNA library preparation

The EV pellet was resuspended in 1X PBS and was treated with RNase A (0.05 μgg/μl) for 30 min at RT. EVs were repurified, and total RNA was isolated using the mirVana miRNA Isolation Kit (Invitrogen). Total RNA input was normalized to the lowest sample concentration. Libraries were made according to the manufacturer’s directions for the TruSeq Small RNA Sample Preparation Kit (Illumina, RS-200-0012). First, 5′ and 3′ small RNA adaptors were ligated to the RNA and ligated products were reverse transcribed using the Superscript II Reverse Transcriptase (Invitrogen). The reverse transcription products were labeled and amplified by performing 13 cycles of PCR. Libraries were then resolved and excised from a 6% polyacrylamide gel (Invitrogen). Resulting gel size–selected libraries (140 to 165 bp) were checked for quality and quantity using the Bioanalyzer and Qubit 2.0 Fluorometer (Life Technologies). Small RNA sequencing libraries (targeting small RNAs of ~22 to 30 nt) were generated from total RNA, as assessed using the Agilent 2100 Bioanalyzer. Equal molar libraries were pooled, requantified, and sequenced as 50-bp single reads on an Illumina NextSeq 2000 instrument using RTA and instrument software versions current at the time of processing. For each sRNA condition, three replicates were collected and sequenced.

### Processing of sRNAs and figure generation

Small RNA reads were trimmed using bbduk (BBMap, B. Bushnell B., sourceforge.net/projects/bbmap/) (options: minlength=17 ktrim=r k=23 mink=11 hdist=1 tpe tbo). Reads matching rRNAs were removed also using bbduk (options: k=31 hdist=1). Reads were aligned with bbmap (options: ambig=best minlen=17 maxindel=0 k=8 rcomp=f local=t) (*69*, *70*). Samtools was used to sort and index alignments (*71*). Bedtools was used to identify reads overlapping with features (*72*). Figures were generated with custom R scripts (https://R-project.org/) using the tidyverse (*73*), cowplot (https://wilkelab.org/cowplot/), and ggvenn libraries (https://CRAN.R-project.org/package=ggvenn). Heatmap expression data (Fig. 2C) are pseudocounts of single-cell clusters from regenerating animals (*39*).

### Gene Ontology

GO enrichment was performed on proteins differentially enriched in large and small EVs with at least a log_2_FC of 1.0, using R with the ClusterProfiler package. GO terms were assigned to the *S. mediterranea* genes using Eggnog-mapper and Interproscan.
