# Supplementary material for: Extracellular vesicles mediate stem cell signaling and systemic RNAi in planarians
Source: Sci Adv. 2026 Feb 6;12(6):eady1461. doi: 10.1126/sciadv.ady1461 (PMC12880550; doi:10.1126/sciadv.ady1461)
Supplement: Supplementary file 1 — Figs. S1 to S5 Legends for tables S1 to S6 [file sciadv.ady1461_sm.pdf]

Science Advances
AAAS

Supplementary Materials for

Extracellular vesicles mediate stem cell signaling and systemic RNAi in planarians

Vidyanand Sasidharan *et al.*

Corresponding author: Alejandro Sánchez Alvarado, asa@stowers.org

Sci. Adv. **12**, eady1461 (2026)
DOI: 10.1126/sciadv.ady1461

The PDF file includes:

Figs. S1 to S5
Legends for tables S1 to S6

Other Supplementary Material for this manuscript includes the following:

Tables S1 to S6

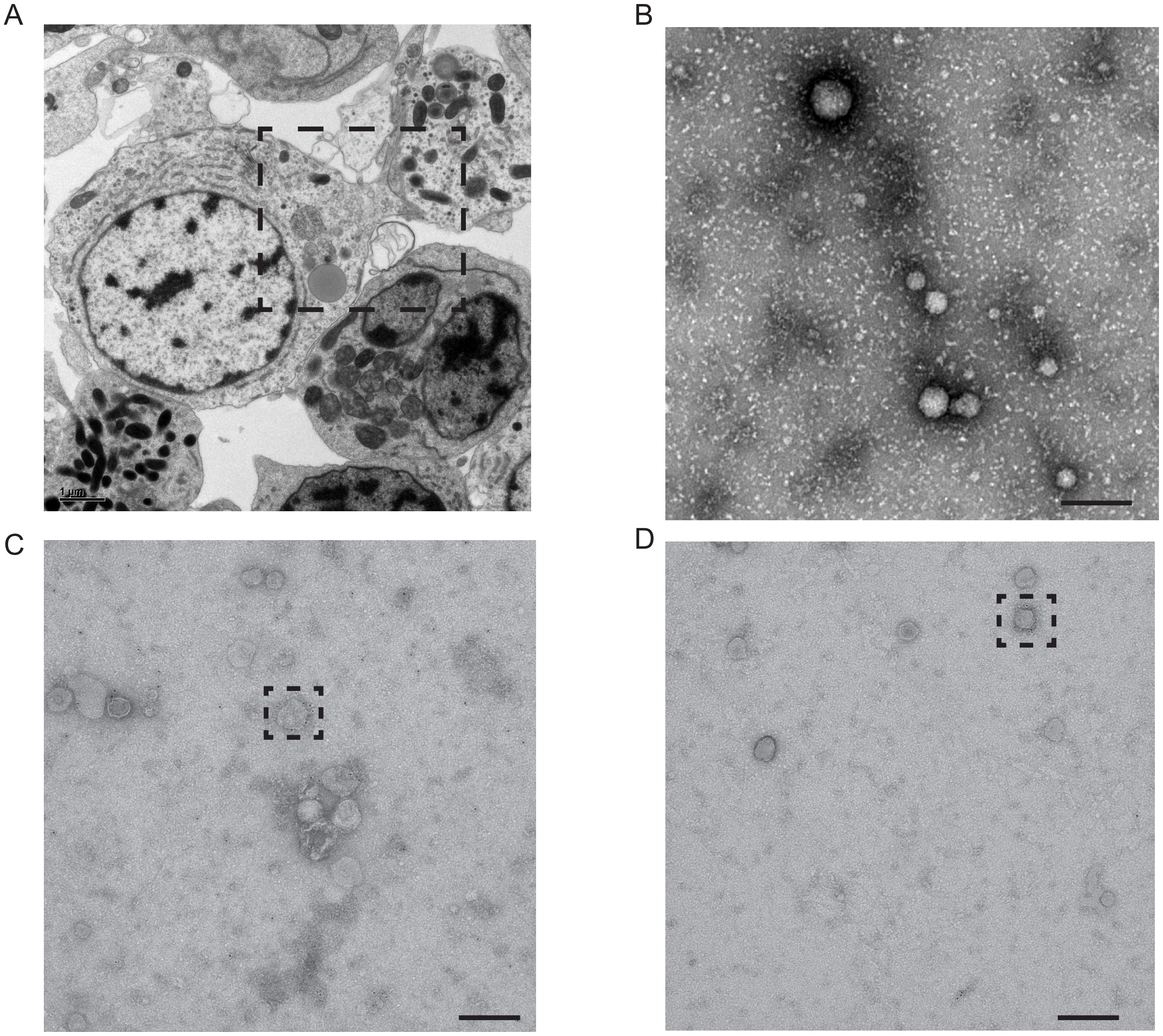

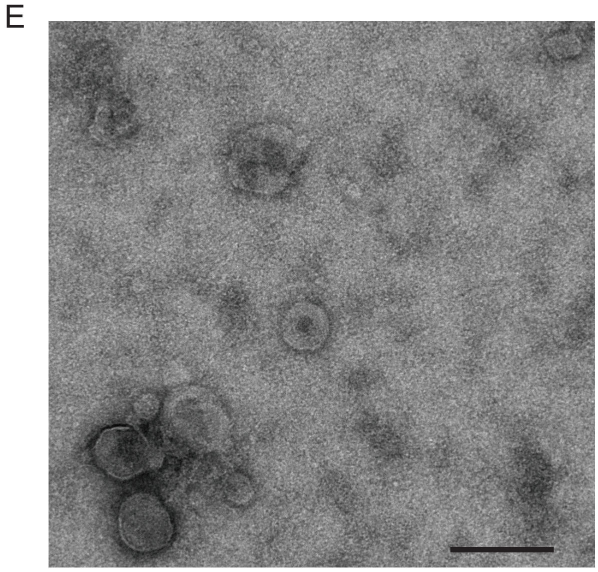

Figure 1 supplement: Presence of EVs in planarian cells. A) A zoomed-out TEM image of 24-hour-cultured X1FS planarian stem cells shows the presence of endosomal vesicles. The dotted black box represents the area shown in Figure 1A. B) EVs isolated from whole animal-rearing culture media. C-D) Immuno gold EM staining on EVs. The black dotted box shows the EVs represented in the main figure. E) No primary antibody control for CD9 and CD63 antibody staining. Scale bar is 100nm.

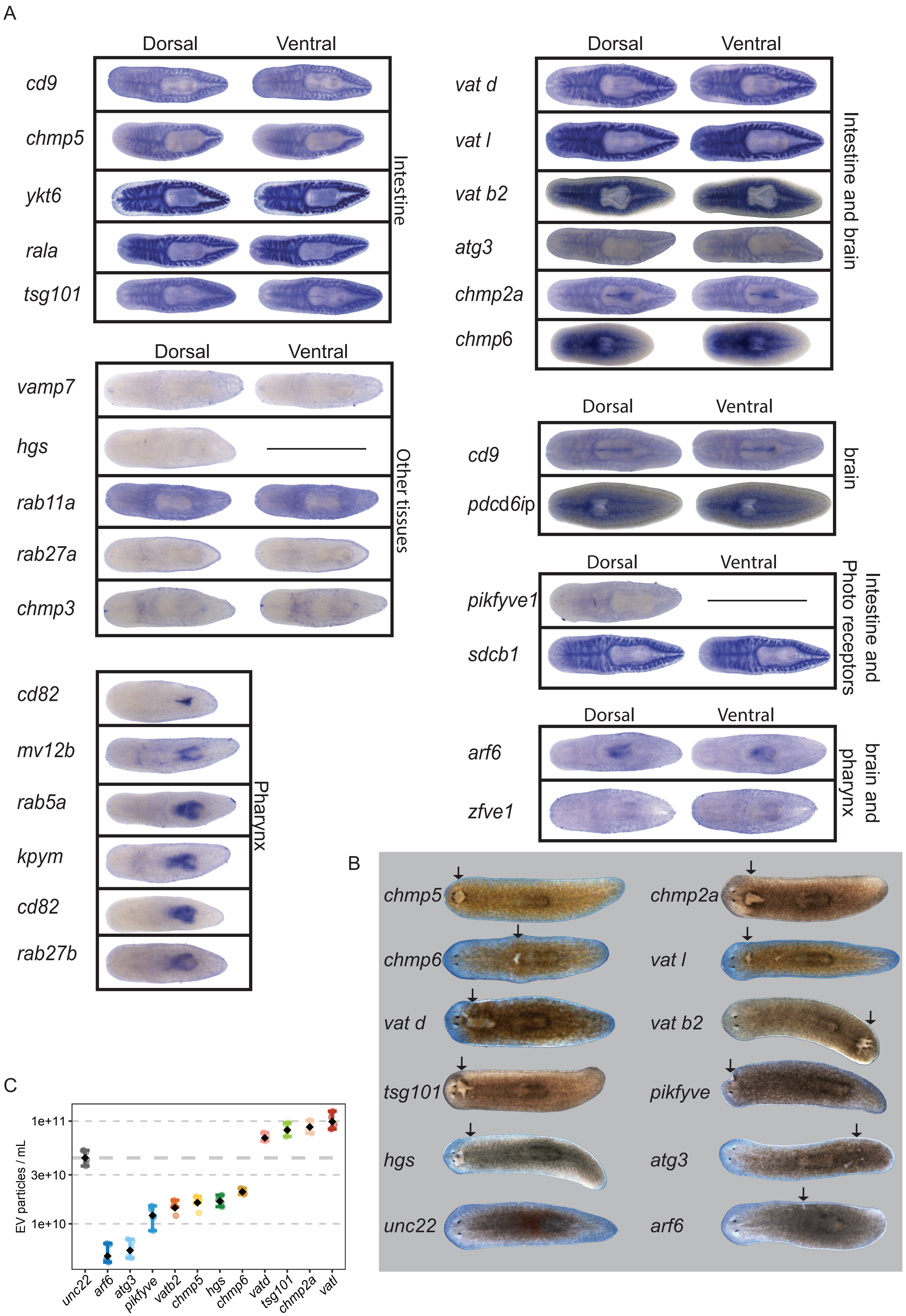
A
Dorsal
Ventral
cd9
chmp5
ykt6
rala
tsg101
Intestine
vat d
vat l
vat b2
atg3
chmp2a
chmp6
Intestine and brain
vamp7
hgs
rab11a
rab27a
chmp3
Other tissues
cd9
pdcd6ip
brain
pikfyve1
sdcb1
Intestine and Photo receptors
cd82
mv12b
rab5a
kpym
cd82
rab27b
Pharynx
arf6
zfve1
brain and pharynx
B
chmp5
chmp2a
chmp6
vat l
vat d
vat b2
tsg101
pikfyve
hgs
atg3
unc22
arf6
C
EV particles / mL
1e+11
3e+10
1e+10
unc22
arf6
atg3
pikfyve
vatb2
chmp5
hgs
chmp6
vatd
tsg101
chmp2a
vatl

Figure 2 supplement: Whole-mount in situ hybridization of ESCRT complex and associated genes. A) WISH identified different mRNA expression patterns in ESCRT complex and associated genes. B) Representative images of RNAi-treated animals. Black arrows indicate epithelial lesions following RNAi. Scale bar- 500 microns. C) Modulation of EV production by members of the ESCRT complex and associated genes upon RNAi treatment (n=200 animals per RNAi). NTA was performed to measure the production of EVs from different knockdown conditions. Black diamonds indicate mean concentrations, whiskers indicate a 95% confidence interval for the mean, and dots represent technical replicates.

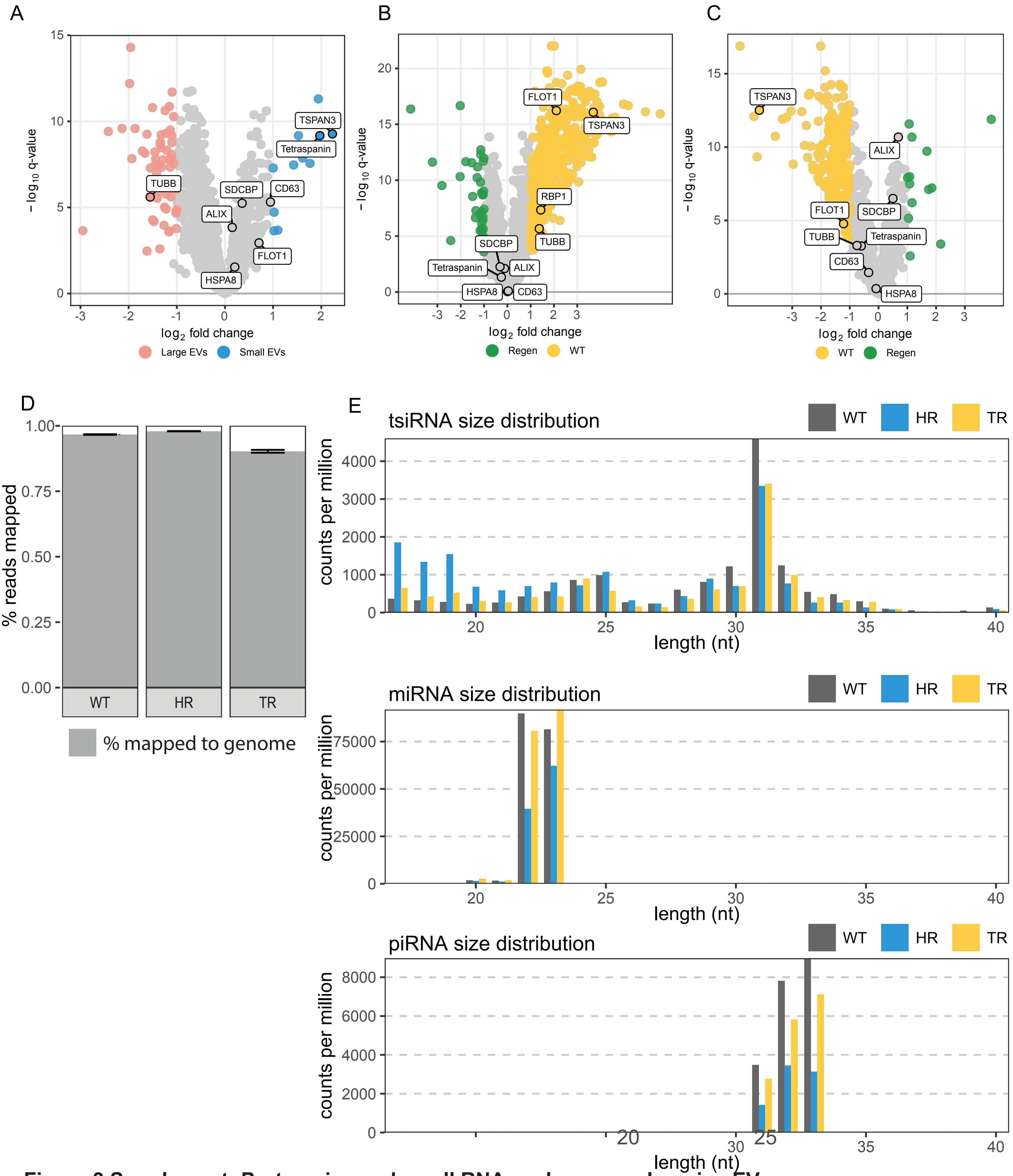

Figure 3 Supplement: Proteomics and small RNA analyses on planarian EVs.
A-C) Differential expression of proteins associated with EVs. A comparative study of proteins related to large (>200nm) and small (<200nm) EVs isolated from wildtype and regenerating animals. A) Comparison of proteins in large and small EVs from regenerating animals. Blue indicates proteins more abundant in large EVs, pink indicates proteins more abundant in small EVs. B-C) Comparison of proteins found in small (B) or large (C) EVs, with green indicating proteins higher in EVs from regenerating worms and yellow indicating proteins higher in wildtype EVs. D) Curated sRNA genome mapping percentage between different experimental conditions. E) Size of major sRNA classes such as tsiRNAs, miRNAs, and piRNAs, across different experimental conditions (WT, HR, and TR). Each plot displays the length (nt) of sRNA fragments and their relative abundance.

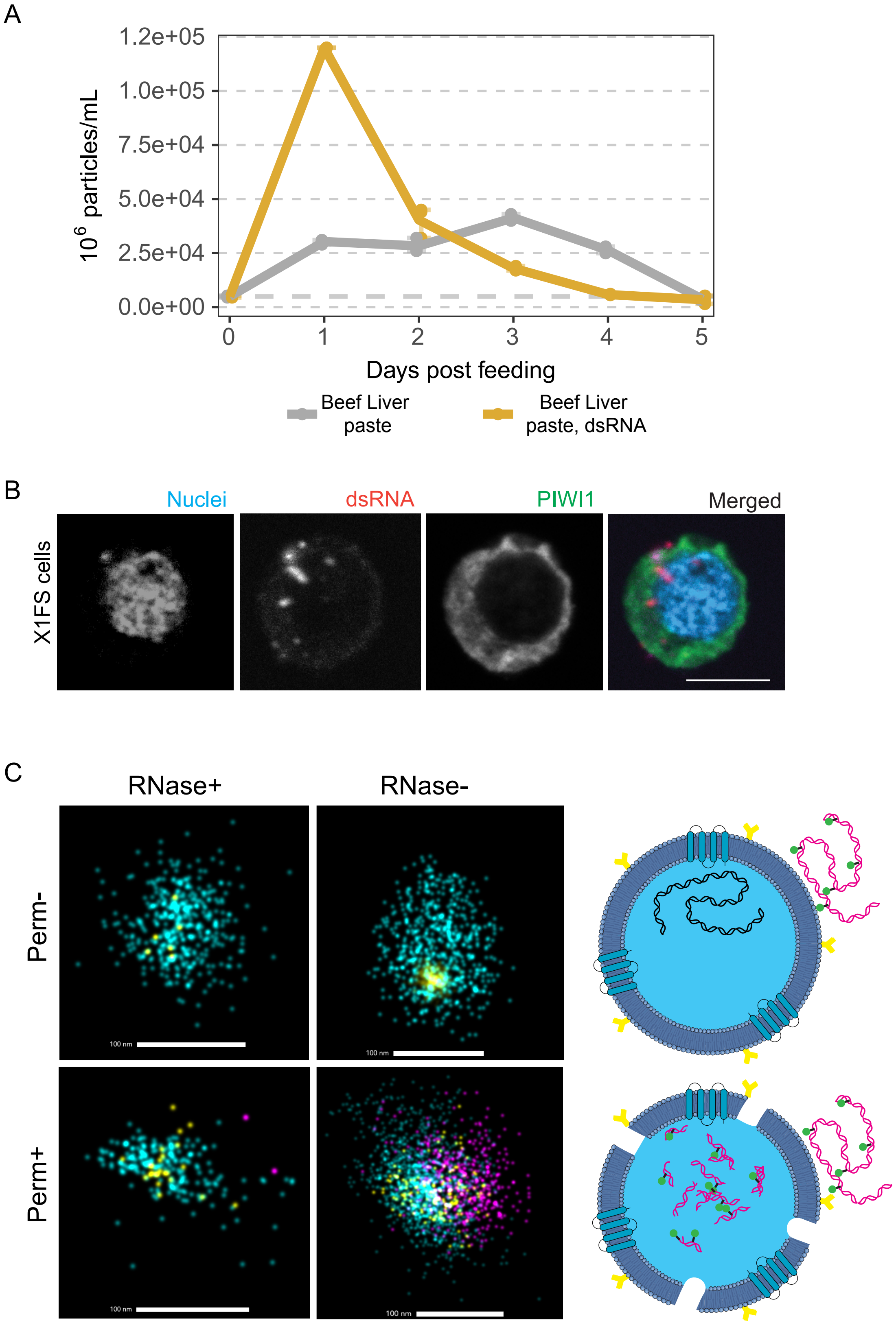

Figure 4 Supplement: Large EVs release and dsRNA detection.

A) NTA quantification of large EV particle production of control and unc22(dsRNA)-fed animals for 5 days post-feeding. Baseline “0-day post feeding” values are from unfed animals. Points represent tech-nical replicates, whiskers indicate a 95% confidence interval for the mean, and both the 95% confi-dence interval and p-value are from a two-sample t-test for fed versus dsRNA fed animals at a given time point. B) Localization of dsRNA inside X1 FS cells. Animals were fed DIG-labeled dsRNA, and X1 FS cells were isolated using cytometry after 2-dpf. Cells were allowed to attach to the plates, and immune staining was performed using anti-DIGOXIGENIN antibody. C) Representative dSTORM images of labeled dsRNA in association with EVs. Cyan- EV membrane dye, Yellow- Pan anti-human tetraspanin (CD9, CD63, CD81) antibody, and magenta-labeled dsRNA.

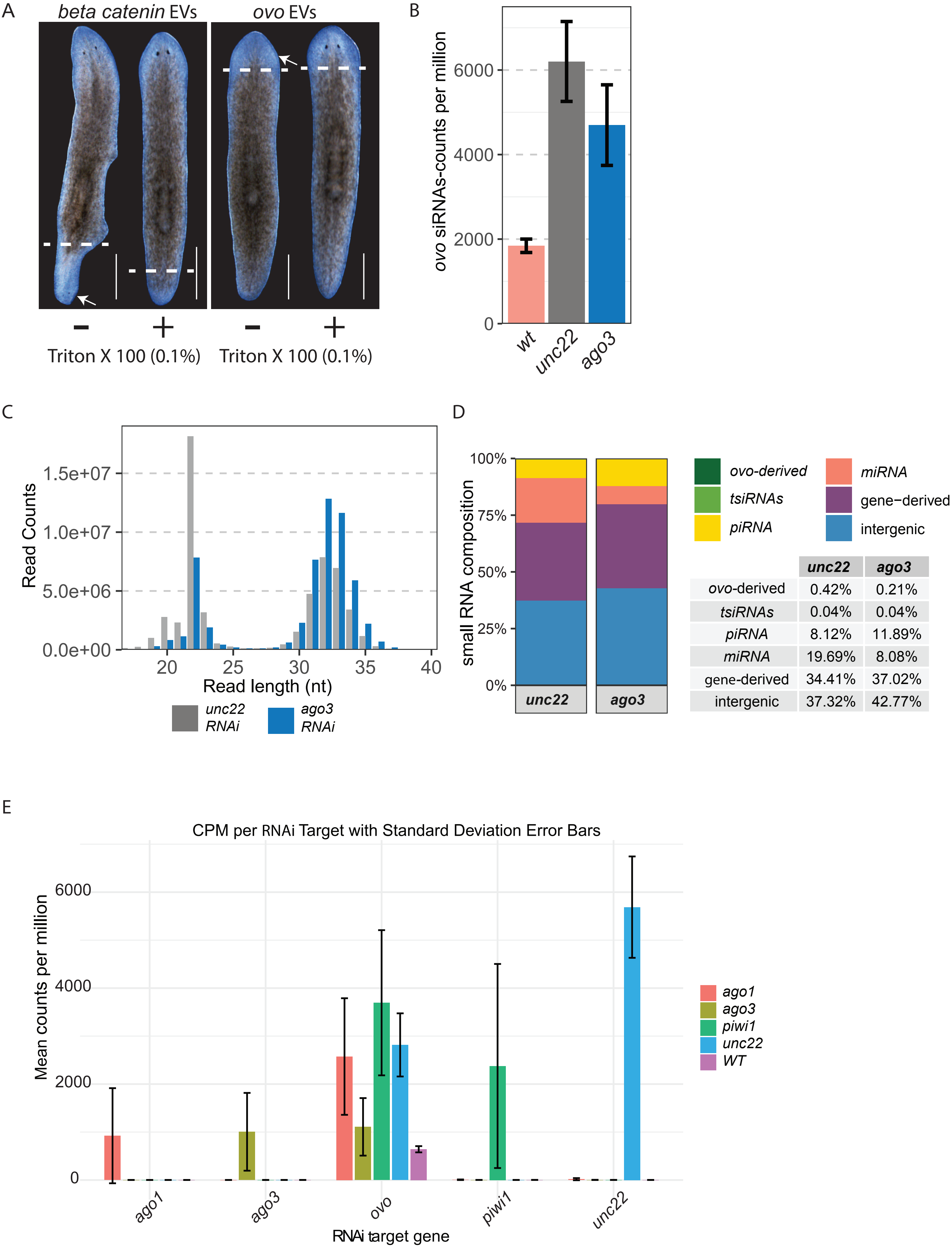

A
beta catenin EVs
ovo EVs
-
+
Triton X 100 (0.1%)
-
+
Triton X 100 (0.1%)
B
ovo siRNAs-counts per million
0
2000
4000
6000
wt
unc22
ago3
C
Read Counts
0.0e+00
5.0e+06
1.0e+07
1.5e+07
20
25
30
35
40
Read length (nt)
unc22 RNAi
ago3 RNAi
D
small RNA composition
0%
25%
50%
75%
100%
unc22
ago3
ovo-derived
tsiRNAs
piRNA
miRNA
gene−derived
intergenic
	unc22	ago3
ovo-derived	0.42%	0.21%
tsiRNAs	0.04%	0.04%
piRNA	8.12%	11.89%
miRNA	19.69%	8.08%
gene-derived	34.41%	37.02%
intergenic	37.32%	42.77%
E
CPM per RNAi Target with Standard Deviation Error Bars
Mean counts per million
0
2000
4000
6000
ago1
ago3
ovo
piwi1
unc22
RNAi target gene
ago1
ago3
piwi1
unc22
WT

Figure 5 Supplement: Quantification of dsRNA-derived siRNAs upon ago gene knockdown. A) Transplantation of permeabilized versus non-permeabilized EVs. Beta catenin EVs and ovo EVs were permeabilized, repurified using ultracentrifugation, and transplanted into healthy animals. Permeabilized EVs did not exhibit any phenotypes(10/10 in both conditions), whereas non-permeabilized EVs (beta catenin EVs-8/10 and ovo EVs-7/10) exhibited gene-specific phenotypes. B) Quantification of ovo(dsRNA)-derived siRNAs associated with tissue from wild type (left), unc-22(RNAi) (middle), and ago-3(RNAi) animals (right). Whiskers represent a 95% confidence interval calculated as 1.96 times the standard deviation above and below the mean. Global processing of dsRNA into siRNAs was not significantly affected upon ago-3 (RNAi). C) Mean percentage of sRNAs by sequence read length(bp) in EVs isolated from ago3(RNAi) and unc22(RNAi) animals. D) Stacked bar plots of the fraction of different sRNA classes (tsiRNAs,miRNAs,piRNAs, gene-derived and intergenic in unc22 and ago3-RNAi conditions. E) siRNAs of non-target genes in dsRNA-treated animals. Bar represents the standard deviation, n=3.

Table legends

Table 1: Proteomics and sRNA counts

Tab1- Proteins enriched in Large EVs and Small EVs. Tab 2- Genome alghment of sRNAs from HR and TR derived EVs. Tab 3- comparison of distinct sRNAs from HR and TR derived EVs.

Table 2: Proteomics

Tab1- A) Comparison of proteins in large and small EVs from regenerating animals. Tab2 & 3) Comparison of proteins found in small (2) or large (3) EVs, with green indicating proteins higher in EVs from regenerating worms and yellow indicating proteins higher in wildtype EVs.

Table 3: GO annotation of EVs

Table 4: WT, HR, and TR-derived EVs trimmed sequences, and non-canonical sRNA reads

Table 5: sRNA classes and counts per replicate for HR, TR, and WT-derived EVs

Table 6: ovo- siRNAs trimmed sequences and non-canonical sRNA reads
